# Electrosensitive Heterogeneous Short Fibers via Acousto‐Electric Coupling for Sequential Bone Regeneration in Infectious Defects

**DOI:** 10.1002/advs.202514174

**Published:** 2025-10-13

**Authors:** Xiaoyu Han, Fan Wang, Pengcheng Xiao, Zheng Yang, Mingyue Liu, Zeyu Han, Zijie Wang, Anan Jiang, Jindong Tan, Juan Wang, Wenguo Cui, Dingqun Bai

**Affiliations:** ^1^ Department of Rehabilitation Medicine, Key Laboratory of Physical Medicine and Precision Rehabilitation of Chongqing Municipal Health Commission The First Affiliated Hospital of Chongqing Medical University No.1 Youyi Road, Yuzhong District Chongqing 400016 P. R. China; ^2^ Department of Orthopaedics, Shanghai Key Laboratory for Prevention and Treatment of Bone and Joint Diseases, Shanghai Institute of Traumatology and Orthopaedics Ruijin Hospital Shanghai Jiao Tong University School of Medicine 197 Ruijin 2nd Road Shanghai 200025 P. R. China; ^3^ State Key Laboratory of Ultrasound in Medicine and Engineering Chongqing Medical University Chongqing 400016 P. R. China

**Keywords:** acoustic‐electric coupling, bone defect, electro‐sensitivity, osteogenesis, ultrasound

## Abstract

The disruption of dynamic equilibrium between antimicrobial and osteogenic processes, caused by the heterogeneous electro‐sensitivity of bacteria and host cells, is central to the high failure rate in repairing infected bone defects. This study collected clinical data and systematically analyzed the limitations of electrical stimulation in bone repair. Consequently, electrosensitive heterogeneous short fibers are innovatively developed, achieving sequential regeneration of infected bone defects through acousto‐electric coupling. First, barium titanate nanoparticles with excellent piezoelectric properties are synthesized by ion substitution doping (BaTiO_3_@Fe). Next, the catechol groups of polydopamine served as multifunctional anchors for the in situ deposition of “conductive” graphene oxide and “piezoelectric” BaTiO_3_@Fe onto short fibers, facilitated by *π*–*π* conjugation and coordination interactions, resulting in the formation of 3D integrated electrosensitive heterogeneous short fibers. At an ultrasound intensity of 1.5 W cm^−^^2^, the system efficiently activates bacterial peroxisome and necroptosis pathways, promoting bacterial apoptosis. At a lower intensity of 0.5 W cm^−^^2^, it activates the TRPV4/Ca^2^⁺/YAP signalling axis, enhancing the osteogenic differentiation of bone marrow‐derived mesenchymal stem cells. By employing a spatiotemporal differential electrical regulation strategy, this coupling approach effectively cascades antimicrobial and osteogenic effects, restoring the electro‐microenvironment homeostasis of bone tissue and significantly accelerating the repair of infected bone defects.

## Graphical abstract

Electrosensitive heterogeneous short fibers can accurately respond to ultrasonic field regulation through the synergistic interaction of piezoelectric and conductive dual elements. At an ultrasound intensity of 1.5 W cm^−^^2^, it can effectively activate bacterial peroxisome and necrotic apoptotic pathway and promote bacterial apoptosis. At 0.5 W cm^−^^2^ it activates the TRPV4/Ca^2^ /YAP axis and enhances osteogenic differentiation of the BMSC.

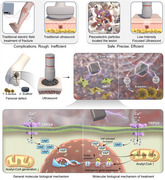

## 1 Introduction

Infectious bone defects represent a severe clinical challenge, characterized by necrosis, collapse, or extensive loss of bone tissue due to infection.^[^ ^1^, ^2^ ^]^ This condition significantly delays bone regeneration and repair and may even pose a life‐threatening risk to the patient. During the progression of infection, bacteria disrupt the bone tissue microenvironment by forming biofilms and secreting toxic factors.^[^ ^3^ ^]^ Concurrently, they activate the host immune response, leading to the release of large quantities of pro‐inflammatory cytokines, which further exacerbate local tissue damage and bone resorption.^[^ ^1^ ^]^ From an osteogenic perspective, bacterial infections inhibit the proliferation and differentiation of osteoblasts while also promoting bone loss by activating bone‐resorbing cells, such as osteoclasts.^[^ ^4^, ^5^ ^]^ Additionally, bacterial metabolites and toxins may disrupt the dynamic balance between osteoblasts and osteoclasts, further impairing bone repair mechanisms.^[^ ^6^ ^]^ Current clinical treatment strategies for infectious bone defects typically involve bone grafting combined with antibiotic therapy.^[^ ^7^ ^]^ However, the overuse of antibiotics not only contributes to the growing problem of antibiotic resistance but also disrupts osteogenic homeostasis during the prolonged treatment process. This disruption compromises the osteogenic potential of bone grafts, ultimately leading to bone repair failure and non‐union of fractures.^[^ ^8^, ^9^ ^]^ Therefore, it is imperative to explore innovative therapies that integrate both antimicrobial and osteogenic capabilities, facilitating osteogenesis following effective infection control and maintaining bone homeostasis. Such innovations are crucial for addressing the high failure rate associated with the continuous repair of infectious bone defects.

Natural bone tissue, as an electrosensitive material, relies on its intrinsic piezoelectric properties to maintain physiological homeostasis. These properties generate endogenous electrical signals through shear force‐induced misalignment and polarisation of collagen fibers, which consequently influence cellular behavior.^[^ ^10^ ^]^ Endogenous electrical signals primarily act by depolarising the membrane potential of osteoblasts, regulating ion channel activity, and promoting transient calcium ion influx into the cells. This cascade activates signalling pathways associated with osteoblast proliferation, differentiation, and bone matrix formation, guiding osteoblast chemotaxis and regulating the expression of osteogenic genes to promote bone repair.^[^ ^10^, ^11^, ^12^ ^]^ Notably, bacteria, which are microorganisms highly sensitive to electrical stimuli, can also detect subtle changes in electrical signals.^[^ ^13^ ^]^ For bacteria, appropriate electrical signal transmission is essential for key physiological activities such as oxidative phosphorylation, membrane transport, and flagellar movement.^[^ ^14^ ^]^ These signals are directly involved in maintaining bacterial energy metabolism and physiological balance. However, when external electrical signals exceed bacterial tolerance thresholds, they can cause transient perforation or rupture of the bacterial cell membrane, disrupting intracellular and extracellular homeostasis and ultimately leading to bacterial death.^[^ ^15^ ^]^ Due to limitations in precision and controllability, current electrical stimulation struggles to match the differing electro‐sensitivity between bacteria and host cells. As a result, they fail to coordinate the continuous and dynamic balance between antibacterial and osteogenic processes, limiting their therapeutic effectiveness. Currently, most electroactive materials (such as piezoelectric ceramics and conductive hydrogels) rely on static electrical signals – whether pre‐stored charges or constant external voltage stimulation – to regulate cell behavior.^[^ ^16^ ^]^ This inherent limitation of static signals in this context is as follows: First, the static electric field cannot be dynamically adjusted as the infection subsides, resulting in insufficient antibacterial efficacy in the early stage of inflammation (due to the fixed low signal intensity) or potential cytotoxicity in the subsequent osteogenic stage (due to prolonged exposure to high signals). Second, static signals cannot match the dynamic cellular processes after infection. Currently, most electroactive materials (such as piezoelectric ceramics and conductive hydrogels) rely on static electrical signals – whether pre‐stored charges or constant external voltage stimulation – to regulate cell behavior. This inherent limitation of static signals in this context is as follows: First, the static electric field cannot be dynamically adjusted as the infection subsides, resulting in insufficient antibacterial efficacy in the early stage of inflammation (due to the fixed low signal intensity) or potential cytotoxicity in the subsequent osteogenic stage (due to prolonged exposure to high signals). Second, static signals cannot match the dynamic cellular processes after infection. Therefore, developing therapeutic strategies that precisely address the electro‐sensitivity heterogeneity of bacteria and host cells is crucial for the effective treatment of infected bone defects.^[^ ^17^ ^]^ By accurately controlling the intensity and frequency of electrical signals, bacterial infections can be effectively inhibited while simultaneously enhancing the regenerative capacity of bone tissue, thereby accelerating the repair of the defect area.

To achieve accurate control over the intensity and frequency of electrical signals while accounting for the electro‐sensitivity heterogeneity between bacteria and host cells, it is crucial to effectively coordinate antibacterial activity and bone formation throughout the continuous repair process. Researchers have developed functional electrodes to deliver external electrical stimulation to infected bone defects or cells. However, these electrodes often suffer from poor targeting accuracy and low efficiency. Piezoelectric materials, when subjected to dynamic stress either in vivo (e.g., tissue fluid flow) or in vitro (e.g., ultrasonic, magnetic, body motion), can convert mechanical energy into electrical energy, producing a form of “self‐power supply” that stimulates tissue metabolic activities. Despite their potential, the following key limitations remain: (1) They generate electricity but lack inherent conductivity; (2) The efficiency and precision of electrical signal conduction cannot be reliably controlled, leading to a disconnect between early‐stage antibacterial action and subsequent repair process, ultimately resulting in repair failure.^[^ ^18^ ^]^ Ultrasound offers a non‐invasive, controllable, precise, and highly penetrative physical stimulus that can mechanically activate piezoelectric materials through cavitation and microstreaming effects,^[^ ^19^, ^20^, ^21^ ^]^ thereby overcoming the reliance on internal dynamic mechanical forces to trigger signal generation and transmission. Additionally, ultrasound allows for precise control of electrical signals through the modulation of its parameters. The charge separation ability of traditional piezoelectric materials in nitrogen is limited, making it difficult to effectively excite reactants, especially since the efficiency of ultrasonic activation to produce reactive oxygen species is low. In this study, Fe‐doped BaTiO_3_ was used, and iron ions were substituted in the BaTiO_3_ crystal structure to generate lattice defects. These defects facilitate electron‐hole separation under ultrasound, driving water and oxygen molecules to participate in redox reactions and produce reactive oxygen species to destroy bacterial structures.^[^ ^22^, ^23^ ^]^ Therefore, the development of ultrasound‐activated, electrosensitive heterogeneous scaffolds that integrate both power generation and electrical conduction represents a significant advancement in this field. These scaffolds can precisely regulate electrical signal transmission, potentially synchronising antibacterial and osteogenic processes and offering new insights for the clinical diagnosis and treatment of infected bone defects.

**Scheme 1:**
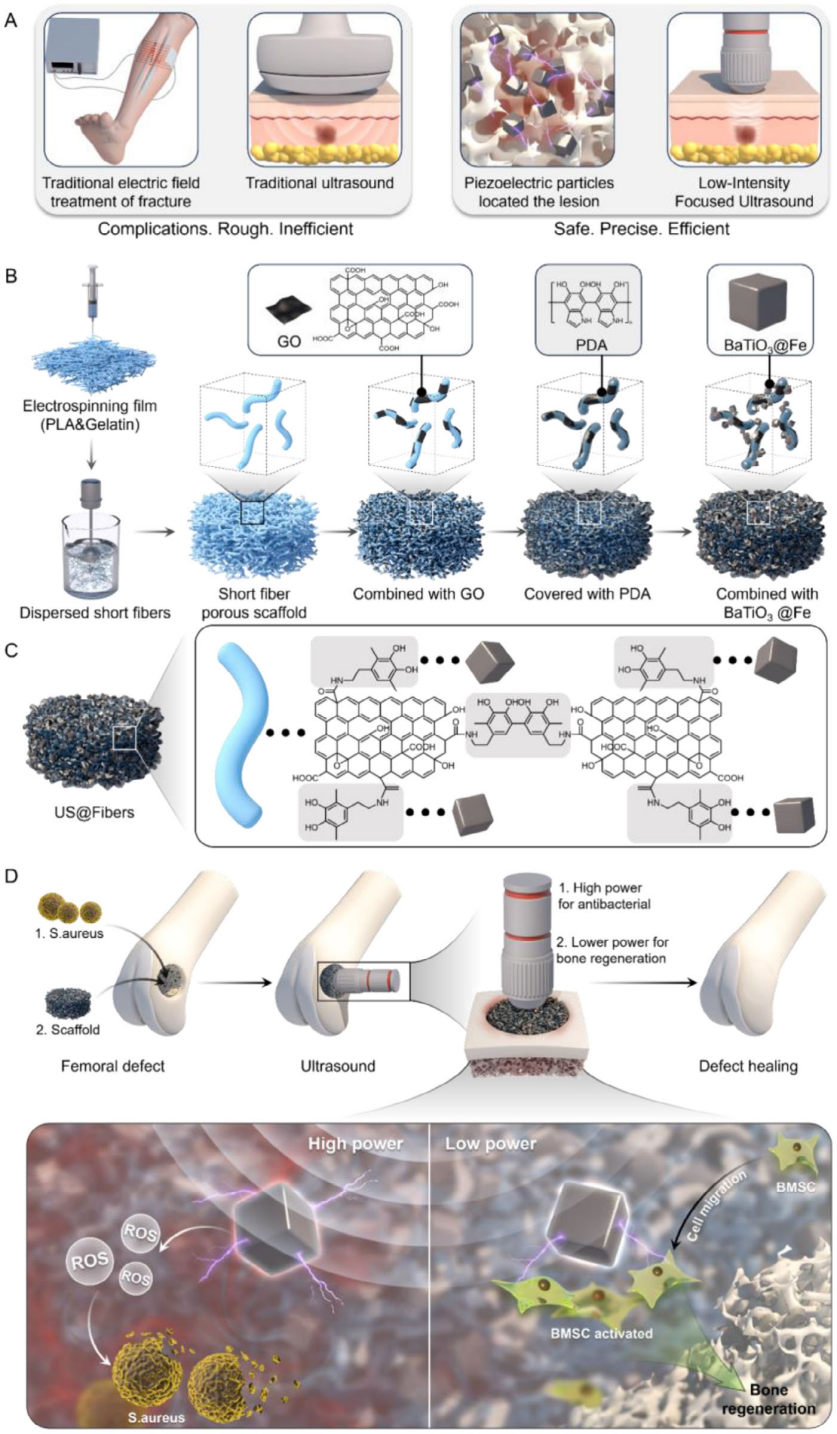
Electrosensitive Heterogeneous Short Fibers via Acoustic‐electric Coupling for Sequential Bone Regeneration in Infectious Defects. A) A comparison pattern diagram of electrical stimulation and ultrasound therapy. B) Synthesis process of Electrosensitive Heterogeneous Short Fibers, C) modification details, and D) in vivo application diagram.

Drawing inspiration from the electrical sensitivity heterogeneity of host cells and bacteria within bone tissue, this study addresses key clinical challenges identified in prior cases. Building upon a previously developed short fiber‐based biomimetic bone tissue extracellular matrix, we report, for the first time, the development of electrically sensitive heterogeneous short fibers, referred to as sono‐electric couplers. By modulating ultrasound frequency in vitro, this approach enables the non‐invasive generation and transmission of multi‐level bioelectrical signals in vivo, allowing for precise regulation of electrical conduction efficiency. At an ultrasound intensity of 1.5 W cm^−2^, the system effectively activates the peroxisome and necroptosis pathways, promoting bacterial apoptosis. At a lower intensity of 0.5 W cm^−2^, it activates the TRPV4/Ca^2+/^YAP axis, promoting osteogenic differentiation of bone marrow‐derived mesenchymal stem cells (BMSCs). This dual‐effect strategy simultaneously achieves sterilisation and osteogenic induction, thereby synchronising with the bone repair process. This study proceeded in two main stages (**Scheme** **1**). First, barium titanate nanoparticles (BaTiO_3_@Fe) were synthesised via ion substitution doping. Second, 3D, piezoelectric‐conductive, integrated electrosensitive heterogeneous short fibers were constructed by in situ deposition of graphene and BaTiO_3_@Fe onto staple fibers, using *π*–*π* conjugations and coordination applying dopamine as an interface coupler. The physicochemical properties of the resulting scaffolds, including their acousto‐electric coupling behavior, morphology, and electrical conductivity, were comprehensively evaluated in vitro. Biocompatibility, cellular infiltration, and antibacterial activity of the scaffolds were also assessed. By establishing an in vivo rat model of infectious bone defect, it was demonstrated that electrosensitive heterogeneous short fibers could regulate multistage bioelectrical signals and accelerate the repair of infectious bone tissue under non‐invasive ultrasound stimulation. Finally, transcriptome sequencing and multi‐omics analyses further elucidated the underlying mechanisms by which the device achieves efficient antibacterial activity while promoting subsequent osteogenesis. In summary, the electrosensitive heterogeneous short fiber, capable of modulating both electrical signal generation and conduction efficiency, offers a novel therapeutic strategy for treating infected bone defects and holds significant promise for clinical translation.

## 2 Results and Discussion

### 2.1 Clinical Electrical Stimulation Therapeutic Effect Analysis and Finite Element Simulation

**Figure 1:**
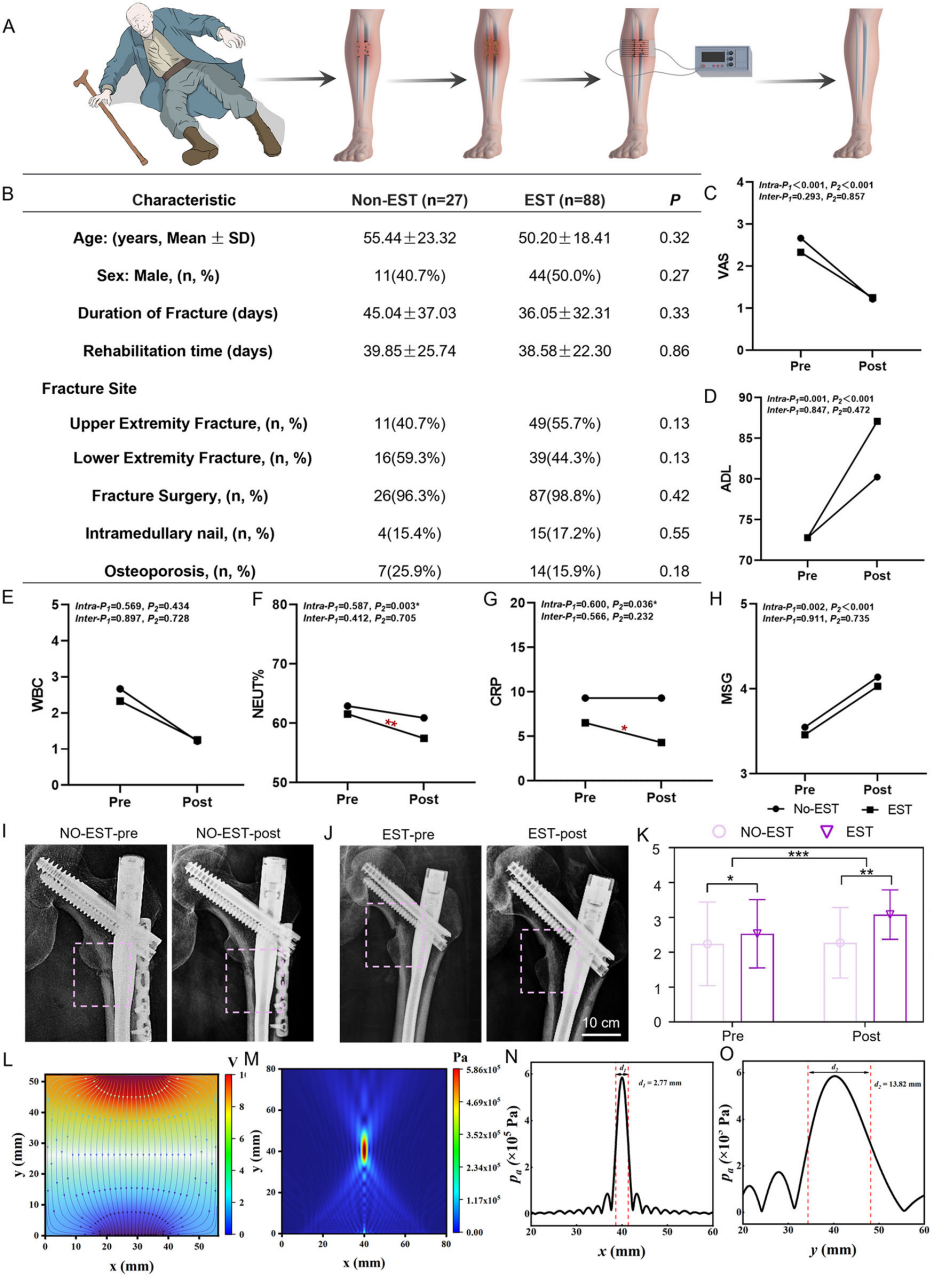
Clinical electrical stimulation therapy and finite element analysis. A) Schematic diagram of clinical electrical stimulation therapy. B) Demographics and clinical characteristics. The comparison of functional scale scores between EST and Non‐EST groups. C) VAS, D) ADL, and H) MSG. The comparison of infection indicators between EST and Non‐EST groups. E) WBC, F) NEUT%, and G) CPR. I, J) The comparison of infection indicators between EST and Non‐EST groups. K) The comparison of FHS between EST and Non‐EST groups. L–O) Finite element simulation of clinical electrical stimulation and focused ultrasound.

In order to systematically analyze the effect of clinical electrical stimulation therapy in the treatment of fractures, we first conducted a clinical randomized controlled study (**Figure** **1**A). A total of 115 patients were included (EST: 88; No‐EST: 27). There were no significant inter‐group differences in age, sex distribution, time from fracture to treatment, overall rehabilitation duration, fracture location (upper vs lower limb), surgical fixation rate, use of intramedullary nails, or presence of concomitant osteoarthritis (P>0.05). Subsequently, a comprehensive comparison was conducted in terms of functional assessment, inflammatory indicators and fracture healing efficacy (Figure 1B) 1. Functional assessments: Both groups demonstrated significant within‐group improvements in VAS, MSG, and ADL scores after rehabilitation (P<0.05), but no statistically significant differences were observed between the EST and No‐EST groups (P>0.05). Notably, the EST group showed a trend toward greater gains in ADL scores (Figure 1C,D,H). 2. Inflammatory Markers: Within the EST group, NEUT% and CRP levels decreased significantly after treatment (NEUT%: P = 0.003; CRP: P = 0.035), whereas WBC counts did not change appreciably. The No‐EST group showed no significant difference between pre‐ and post‐treatment in any inflammatory marker (P>0.05). Between group comparisons of post‐treatment inflammatory values did not reach statistical significance (P>0.05) (Figure 1E–G). 3. Fracture healing: Comparative radiographic analysis in patients with concurrent infections demonstrated that the EST group experienced a significant increase in Fracture Healing Score (FHS) after treatment. The baseline FHS did not differ between groups, post‐treatment FHS was markedly higher in the EST cohort (P = 0.010) (Figure 1K). As shown in Figure 1I,J, radiographs taken 30 days after femoral fracture revealed blurred fracture lines indicating callus formation in the EST group, whereas distinct fracture lines persisted in the No‐EST group. To sum up, electrical stimulation therapy produces improvements in pain management, muscle strength, and functional independence comparable to standard treatment alone. Importantly, electrical stimulation significantly reduces key inflammatory markers in infection‐associated fractures and accelerates Fracture healing. These findings support the use of electrical stimulation as a noninvasive therapy to promote bone healing and control infection in fractures. Although electrical stimulation has shown significant therapeutic effects in treating infection‐related fractures, long‐term clinical observation data have revealed instability in its treatment outcomes. To explore the reasons for this instability in clinical electrical stimulation therapy, this study employed a finite element simulation method for analysis. The results showed that while electrical stimulation therapy can conduct electrical signals to the target treatment area, it has significant shortcomings in the accuracy and controllability of the signals. In contrast, low‐intensity focused ultrasound technology has demonstrated unique advantages. It can effectively penetrate the skin, connective tissue, and muscular layers of the bone surface, and accurately regulate the affected area. Its focusing precision reaches 2.77 mm along the X‐axis and 13.82 mm along the Y‐axis, which is sufficient to meet the needs of precise treatment for infectious fractures in different locations (Figure 1L–O). Based on these findings, we innovatively developed an “acoustic‐electric coupler.” This device aims to leverage the advantages of electrical signal therapy while utilizing the precision and superior penetration of ultrasound. By converting acoustic and electrical signals, it enables non‐invasive external regulation of electrical signals in the affected area, thereby effectively promoting the healing process of infected bone defects and providing a more precise and efficient new method for the treatment of infected fractures.

### 2.2 Fabrication and Characterization of the Acoustic‐Electric Couplers

**Figure 2:**
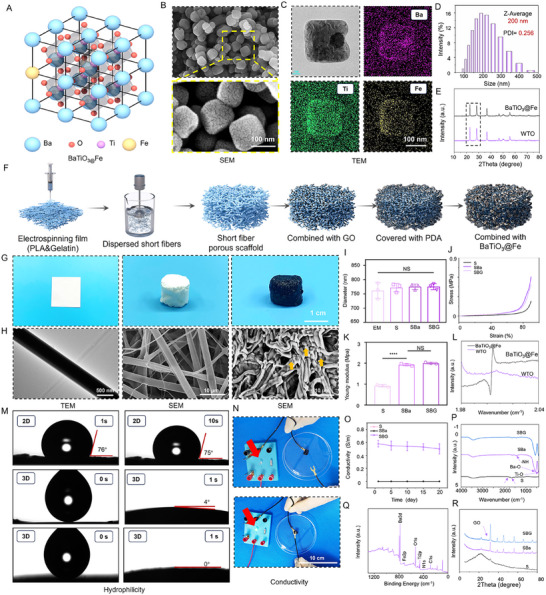
Characterization of Acoustic‐electric Couplers. A) Schematic illustration of the structure of barium titanate. B) SEM image of barium titanate. C) EDS elemental analysis. D) Particle size distribution of BaTiO_3_@Fe nanoparticles. E) XRD pattern. F) Schematic diagram of the preparation of acoustic‐electric couplers. G, H) Digital camera images, SEM, and TEM images of the composite short fiber scaffold. Yellow arrows denote BaTiO_3_ nanoparticles. I, J, K) Diameter of the composite short fiber scaffold, stress–strain curve, and Young's modulus. L) EPR spectrum. O, N) Conductivity images of the composite short fiber scaffold. P) FTIR spectrum. Q) XPS spectrum. R) XRD pattern. M) Contact angle images of the composite short fiber scaffold.

The acoustic‐electric couplers (US@Fibers), an implantable device, primarily comprises piezoelectric components of barium titanate, functioning as a “power‐generating” nanogenerator. It converts ultrasonic mechanical force into electrical signals to regulate local tissue activity. The acoustic‐electric couplers are fabricated from PLA and gelatin, with graphene oxide added to enhance conductivity.^[^ ^24^, ^25^ ^]^ These components are integrated into a 3D piezoelectric‐conductive bioactive short fiber via polydopamine (PDA) redox reactions and multifunctional catechol groups. To confirm the successful fabrication, we first characterized the piezoelectric part. Tetragonal‐phase piezoelectric nanoparticles, chosen for their high piezoelectric effect, efficient acoustic‐electric coupling, and good biocompatibility, show great potential in bone repair, nerve regeneration, and immune microenvironment modulation. In this experiment, BaTiO_3_@Fe (iron‐doped barium titanate) was synthesized hydrothermally (**Figure** **2**A). In this study, TEM, SEM, and elemental mapping were employed to investigate the distribution and surface morphology of BaTiO_3_@Fe. SEM analysis revealed a typical tetragonal phase morphology with excellent piezoelectric properties, characterized by uniform and stable size distribution. Elemental mapping demonstrated the presence of Ba, Fe, and Ti, confirming the tetragonal phase structure (Figure 2B,C). Dynamic light scattering further elucidated the particle size distribution of BaTiO_3_@Fe, showing a uniform, monomodal distribution with an average particle size of 200 nm and a polydispersity index (PDI) of 0.256 (Figure 2D). To characterize the tetragonal crystal structure of BaTiO_3_@Fe, X‐ray diffraction analysis indicated that the diffraction peaks matched the standard PDF card for tetragonal barium titanate (Figure 2E). Notably, a double‐peak structure observed at ≈2θ = 50° is a characteristic feature of tetragonal barium titanate, confirming the excellent piezoelectric properties of the synthesized nanoparticles. Similarly, Figure 2L showed a distinct oxygen vacancy peak in the 2–2.02 wavenumber (cm^−1^) range, attributed to the binding of Fe^2^⁺ ions with oxygen atoms. Figure 2F illustrates the synthesis process of the acoustic‐electric couplers. Figure 2G depicts the synthesis process of the acoustic‐electric couplers, transitioning from a 2D fiber membrane to a 3D short fiber scaffold, as evidenced by digital camera images and subsequent TEM and SEM images (Figure 2H). The yellow arrows point to barium titanate nanoparticles. This distribution ensures that barium titanate can effectively convert ultrasonic energy into electrical signals through the piezoelectric effect, laying the foundation for the electrical sensitivity of the fibers. Additionally, Figure 2I illustrates the modification process from a 2D to a 3D structure. However, the nanofiber diameter did not change statistically significantly, despite a slight increase. The high surface area and microstructure of the nanofibers were preserved, allowing for enhanced interaction with cell membranes, thereby promoting cell adhesion and signaling, and accelerating cell growth, proliferation, and differentiation. This surface structure is capable of regulating cell behavior at the nanoscale, which is particularly critical for the differentiation of BMSCs and ultimately contributes to improved tissue repair efficiency.^[^ ^26^ ^]^

To further characterize the successful synthesis of the acoustic‐electric couplers, chemical composition analysis was performed using FTIR, XRD, and XPS. As shown in Figure 2P, characteristic peaks of the ester carbonyl groups from polylactic acid and the carbonyl groups from the polypeptide chains in gelatin were observed between 2000 and 1500 cm^−1^, confirming their integration into the fiber scaffold. The bending vibrations of the amine groups (−NH) in polydopamine were detected between 1500 and 1250 cm^−1^. Peaks corresponding to the bending vibrations of Ba−O and Ti−O near 500 cm^−1^ confirmed the successful grafting of BaTiO_3_@Fe onto the fiber scaffold via polydopamine. Since graphene cannot be detected by infrared analysis, XRD was employed as a complementary validation tool. As shown in Figure 2R, the characteristic peak of graphene was observed at 2θ = 30°, confirming its incorporation into the fiber scaffold. Additionally, the characteristic peaks of nitrogen, titanium, and barium observed in the XPS results (Figure 2Q; Figure S1, Supporting Information) further confirmed the successful fabrication of the acoustic‐electric couplers. To meet the requirements of an implantable acoustic‐electric couplers, the mechanical properties of the scaffold were evaluated. Figure 2J,K demonstrate that after modification with BaTiO_3_@Fe and graphene oxide, the Young's modulus of the scaffold met the mechanical demands for infectious bone defects. Notably, the piezoelectric‐conductive bioactive fiber scaffold exhibited excellent hydrophilicity, which is essential for capturing biological information from local tissues and integrating the piezoelectric and conductive functions effectively. As shown in Figure 2M, the contact angle between a water droplet and the 2D fiber membrane was 76° at 1 s and 75° at 10 s, indicating poor water absorption. In contrast, the 3D fiber scaffold absorbed the water droplet within 1 s, resulting in a contact angle of 4° or 0°, demonstrating rapid absorption. This suggests that the bioactive fiber scaffold can efficiently capture biological information from local tissues, thereby laying the foundation for promoting bone tissue repair. The binding affinity of PDA between GO and the scaffold: Polydopamine has a wide range of adhesion capabilities and can form stable coatings on various organic and inorganic material surfaces. It is one of the most commonly used surface functionalization modification methods.^[^ ^27^, ^28^ ^]^ In this study, PDA acts as an adhesive, and its adhesive performance mainly stems from multiple synergistic mechanisms: First, the hydrophilic groups such as phenolic hydroxyl groups (—OH) and amino groups (—NH_2_) abundant in PDA can form hydrogen bonds and electrostatic adsorption with the substrate scaffold, firmly adhering to the scaffold surface. Second, the aromatic amine structure in PDA forms *π*–*π* conjugation with the carbon skeleton of GO. Additionally, PDA can form stable chelate structures with metal ions (such as Ba^2^⁺, Ti⁴⁺, Fe^3^⁺) on the surface of BaTiO_3_@Fe through metal‐coordination complexation, further enhancing the fixation ability of inorganic components. Through the synergistic action of multiple adhesion mechanisms, PDA can firmly connect the organic scaffold with inorganic nanoparticles. In summary, the acoustic‐electric couplers developed in this study exhibit excellent piezoelectric, conductive, and hydrophilic properties. These features provide a basis for non‐invasive remote generation and transmission of multi‐level bioelectrical signals within the body, precise regulation of electrical conduction efficiency, and dual effects of antibacterial and osteogenic induction, thus establishing a foundation for innovative therapeutic strategies in tissue repair.

### 2.3 Characterization of the “Power‐Generating” and “Conductive” Properties of the Acoustic‐Electric Couplers

**Figure 3:**
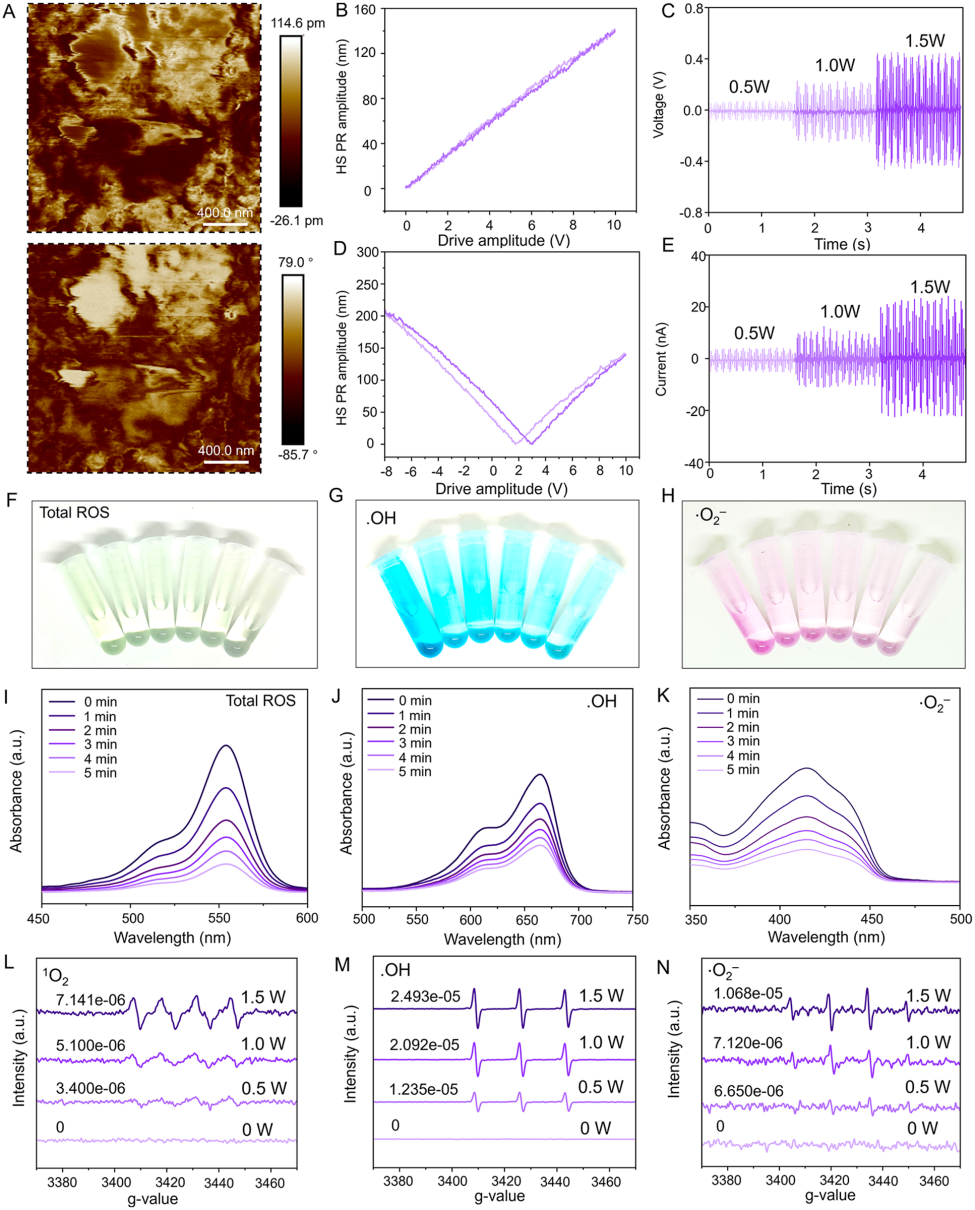
Characterization of Acoustic‐Electric Coupling in Acoustic‐electric Couplers. A) Amplitude and phase images from piezoresponse force microscopy (PFM) analysis. B, D) Butterfly hysteresis loops and d_33_ curves of barium titanate (BaTiO_3_). C, E) OC voltage and SC current of the electroactive short fiber scaffold at different ultrasound powers. F–H) Photographic images of ROS generation by acoustic‐electric couplers degrading rhodamine B (RhB), methylene blue (MB), and 1,3‐diphenylisobenzofuran (DPBF) at different ultrasound intensities. I–K) Absorbance measurements corresponding to the degradation of RhB, MB, and DPBF by acoustic‐electric couplers at different ultrasound intensities. L–N) Electron spin resonance (ESR) spectra quantifying the production of ^1^O_2_, •OH, and •O_2_^−^ by acoustic‐electric couplers at different ultrasound intensities.

The acoustic‐electric couplers are designed to treat infectious bone defects by precisely controlling the intensity and frequency of electrical signals, effectively integrating antibacterial and osteogenic processes into a continuous repair cascade. The integration of conductive and power‐generating functions is crucial for achieving this continuous repair. This section validates the conductivity and power‐generating capabilities of US@Fibers. First, regarding conductivity, the disruption of local bioelectrical signal transmission and the destruction of the bioelectric field are common consequences of infectious bone defects. Therefore, we assessed the conductivity of US@Fibers by integrating the acoustic‐electric couplers into an electrical circuit. As shown in Figure 2N, the light‐emitting diode (LED) remains unlit when not in contact with US@Fibers. However, upon contact, the LED is clearly illuminated, demonstrating that US@Fibers effectively conduct electrical signals during tissue regeneration. This conductivity is essential for recruiting and guiding cells to migrate and promote bone tissue repair. Notably, bone tissue requires stable electrical conductivity to transmit long‐term electrical signals for reshaping the damaged local bioelectric field. Thus, we evaluated the stability of electrical conductivity over 20 days of culture. As shown in Figure 2O, the conductivity of US@Fibers did not significantly decay, confirming that the acoustic‐electric couplers can maintain stability for at least 20 days. Second, the power‐generating capability of US@Fibers is derived from BaTiO_3_@Fe, which converts ultrasonic mechanical signals into electrical signals. These signals help activate ion channels on the cell surface, effectively integrating the continuous repair process following antibacterial treatment in the repair of infectious bone defects. To characterize the power‐generating ability of US@Fibers, piezoresponse force microscopy (PFM) amplitude and phase maps showed good matching of bright and dark regions of BaTiO_3_@Fe under a reversed electric field (**Figure** **3**A). Similarly, butterfly hysteresis loops (Figure 3D) indicated polarization reversal of BaTiO_3_@Fe under an external polarizing electric field, confirming the piezoelectric effect. Furthermore, the piezoelectric constant of BaTiO_3_@Fe was calculated to be ≈14.05 pm V^−1^ based on the d_33_ curve (Figure 3B).

It is well‐known that the integration of antibacterial and osteogenic processes is crucial for the repair of infectious bone defects. Antibacterial treatment not only inhibits the growth of pathogens but also promotes the normal function of the immune system by reducing local inflammatory responses.^[^ ^29^ ^]^ When antibacterial treatment is effectively integrated with the repair process, it accelerates the recruitment of immune cells and the release of bone‐healing factors, enhancing new bone formation and remodeling.^[^ ^30^ ^]^ A prerequisite for this tight integration is the precise control of power generation by the acoustic‐electric couplers, specifically the gradient responsiveness of US@Fibers to ultrasound. To evaluate the acoustic‐electric coupling efficiency of US@Fibers, an oscilloscope was used to measure the open‐circuit voltage and short‐circuit current under ultrasound irradiation. As shown in Figure 3C,E, at an ultrasound power of 0.5 W cm^−^^2^, the open‐circuit voltage (OC voltage) was 85 mV, and the short‐circuit current (SC current) was 3.92 nA. At 1 W cm^−^^2^, the OC voltage was 205 mV, and the SC current was 11.52 nA. At 1.5 W cm^−^^2^, the OC voltage was 450 mV, and the SC current was 22.8 nA. These results demonstrate that US@Fibers exhibit excellent responsiveness to ultrasound, converting acoustic energy into electrical signals across different ultrasound intensities, which is significant for the subsequent integration of antibacterial and repair processes. Notably, the primary antibacterial component of the acoustic‐electric couplers is BaTiO_3_@Fe, a piezoelectric material with an intrinsic non‐centrosymmetric structure. Under ultrasound irradiation, this material deforms to generate an internal electric field, which drives electron‐hole separation and directs their transport to opposite surfaces. In this process, reactive oxygen species (ROS) are produced as a result of redox reactions with oxygen or water molecules. The ROS, including singlet oxygen (^1^O_2_), hydroxyl radicals (•OH), and superoxide anions (O_2_^−^), have strong oxidizing capabilities that can disrupt bacterial cell walls, membranes, and intracellular contents (such as DNA, RNA, and proteins).^[^ ^31^ ^]^ These ROS directly attack bacterial biomolecules, destroying their structure and function, thereby effectively killing bacteria.^[^ ^32^ ^]^ To verify the ROS generation efficiency of US@Fibers, electron spin resonance spectroscopy (ESR) was employed. As shown in Figure 3F–K, the generation of hydroxyl radicals (•OH), superoxide anions (O_2_^−^), and total ROS was confirmed using methylene blue, 3,3',5,5'‐tetramethylbenzidine, and rhodamine B, respectively. The results indicate that the acoustic‐electric couplers have efficient ROS‐generating capabilities. Additionally, as shown in Figure 3L–N, the production of •OH, O_2_^−^, and ^1^O_2_ and their responsiveness to ultrasound were validated. With increasing ultrasound intensity, the concentrations of •OH, O_2_^−^, and ^1^O_2_ also increased. At an ultrasound intensity of 1.5 W cm^−^^2^, the concentrations were 7.141 × 10^−^⁶ for ^1^O_2_, 2.493 × 10^−^⁵ for •OH, and 1.068 × 10^−^⁵ for O_2_^−^. This demonstrates that US@Fibers can effectively generate ROS in response to ultrasound, providing a foundation for antibacterial treatment in infectious bone defects. In terms of response sensitivity, most existing electroactive materials require external high‐voltage stimulation or large‐amplitude mechanical stress to generate effective electrical signals that regulate bone regeneration. In contrast, the heterogeneous short fibers in this study rely on the acoustic‐electric coupling effect and can produce stable electrical responses under low‐intensity ultrasound stimulation (power density 0.5 W cm^−^^2^). This characteristic is closer to the physiological electrical microenvironment of bone tissue and avoids tissue damage that may be caused by high‐intensity stimulation. Moreover, existing electroactive materials generally have limitations, such as poor biodegradability (e.g., piezoelectric ceramics) or low mechanical compatibility with bone. The heterogeneous short fibers in this study have similar mechanical properties to cancellous bone and achieve a synchronous process of “electrical stimulation – bone regeneration”. This unique performance advantage makes the prepared electro‐sensitive heterogeneous short fibers a more promising candidate material in the field of infectious bone defect repair.

### 2.4 Antibacterial Efficacy of the Acoustic‐Electric Couplers

**Figure 4:**
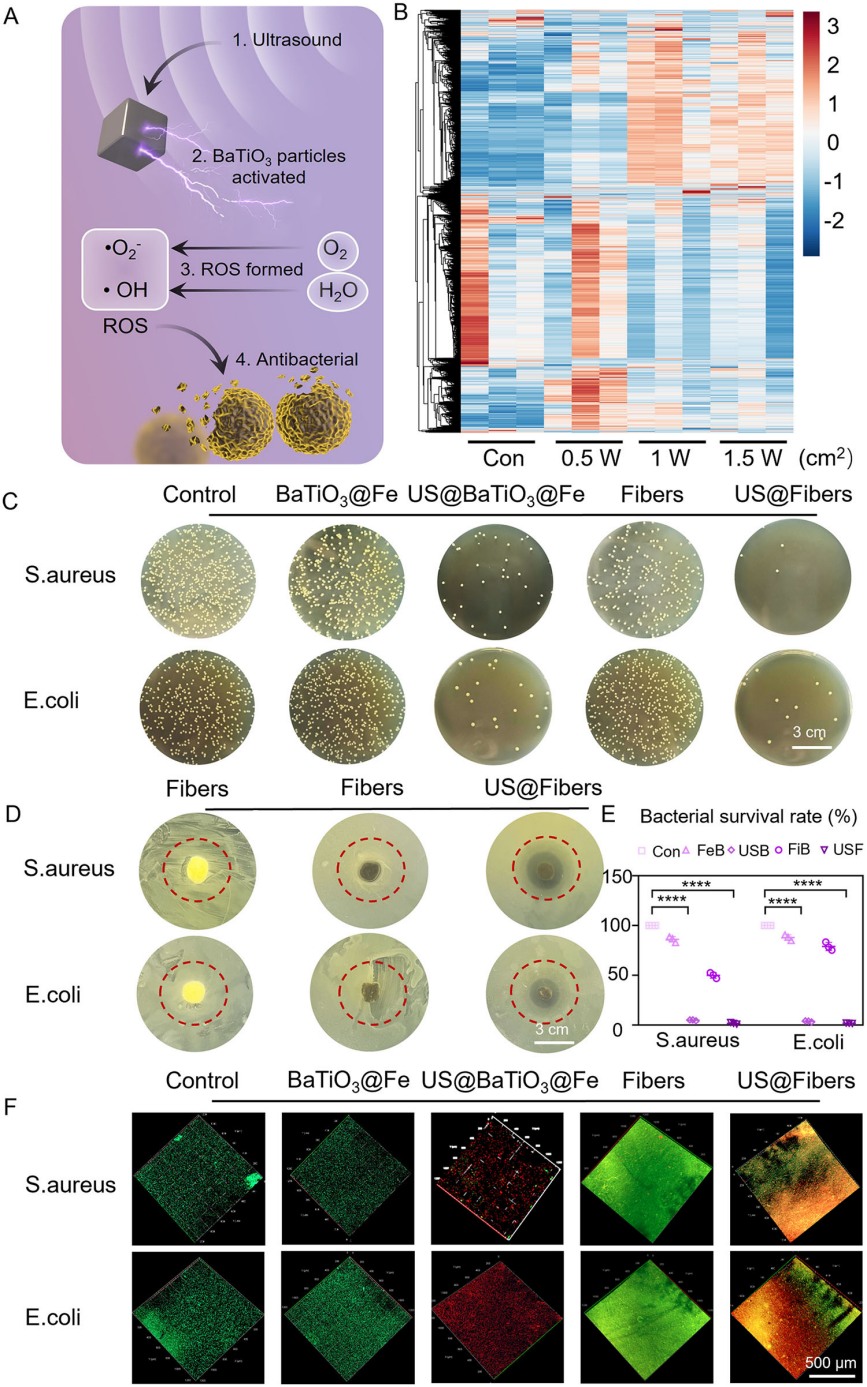
Validation of Antibacterial Efficacy of Acoustic‐electric Couplers. A) Schematic illustration of the antibacterial mechanism of the material. B) Transcriptomic sequencing results demonstrating antibacterial efficacy. C) Antibacterial validation using the spread plate method. D) Zone of inhibition assay for acoustic‐electric couplers. E) Quantitative analysis of the spread plate experiment. F) Live/dead staining images of Escherichia coli and Staphylococcus aureus.

Staphylococcus aureus and Escherichia coli are the most common pathogens in postoperative orthopedic infections.^[^ ^33^ ^]^ After orthopedic surgery, S. aureus can rapidly form biofilms on bone surfaces and implants, creating a physical barrier that hinders antibiotic penetration and evades host immune clearance. The presence of biofilms makes infections difficult to eradicate, inducing a chronic inflammatory environment that impairs the differentiation and function of osteoblasts, thereby delaying bone regeneration.^[^ ^34^ ^]^ Moreover, bacterial toxins secreted by S. aureus and E. coli can directly damage osteoblasts and the bone matrix, leading to local bone resorption. These toxins can also activate immune cells such as macrophages, inducing chronic inflammatory responses and secreting large amounts of pro‐inflammatory cytokines, which enhance osteoclast activity.^[^ ^35^ ^]^ To better integrate antibacterial and osteogenic processes, controlling the infection is essential. The acoustic‐electric coupler generates ROS by deforming the BaTiO_3_@Fe piezoelectric component under ultrasound irradiation. These ROS can disrupt the integrity of bacterial cell membranes, leading to cytoplasmic leakage and cell death (**Figure** **4**A). To verify the antibacterial efficacy and mechanism of the acoustic‐electric couplers, this section employs bacterial standard plate counting, the agar diffusion method, live/dead cell staining, and bacterial transcriptome sequencing. First, we investigated the changes in bacterial transcriptional signals with varying ultrasound intensities through transcriptome sequencing. Four groups were tested: Control, 0.5 W, 1 W, and 1.5 W. The quality control data of the sequencing results are shown in Figure S2 (Supporting Information). As the ultrasound intensity increased, the number of differentially expressed genes in the bacterial transcriptome also increased. Heatmap analysis revealed that the gene expression levels between the 1.5 W group and the Control group showed the greatest differences (Figure 4B). Gene Ontology (GO) enrichment analysis of these differentially expressed genes indicated that the primary biological function changes were related to alterations in molecular binding (Figure S3, Supporting Information). KEGG enrichment analysis was performed on differentially expressed genes between 1 W versus 1.5 W (Cluster 1), 0.5 W versus 1 W (Cluster 2), and Control versus 0.5 W (Cluster 3) (Figures S4–S6, Supporting Information). The results showed that Cluster 1 was enriched in pathways such as Peroxisome, Necroptosis, and Ferroptosis; Cluster 2 was enriched in pathways such as Chemical Carcinogenesis‐Reactive Oxygen Species and Oxidative Phosphorylation; and Cluster 3 was enriched in pathways such as Bacterial Secretion System, Bacterial Chemotaxis, and Exopolysaccharide Biosynthesis. These findings suggest that low‐intensity ultrasound is insufficient to generate enough electrical signals to kill bacteria. However, when the intensity exceeds 1 W, it can produce sufficient ROS to activate bacterial apoptosis, with the highest efficiency observed at 1.5 W. Based on these results, we further validated the antibacterial efficacy of the acoustic‐electric couplers. As shown in Figure 4C, the US@Fibers and US@BaTiO_3_@Fe groups achieved bacterial inhibition rates of 98.30% and 95.16% against S. aureus, respectively, and 98.20% and 96.34% against E. coli, respectively. In contrast, the Fibers and BaTiO_3_@Fe groups, which lacked ultrasound irradiation, showed no significant difference in inhibition rates compared to the Control group, indicating that the acoustic‐electric couplers and their components efficiently generated ROS under ultrasound irradiation, disrupting bacterial metabolism and causing cell membrane rupture. To further confirm the antibacterial efficacy of the acoustic‐electric couplers, the agar diffusion method was employed as a second experimental approach. As shown in Figure 4D; Figure S7 (Supporting Information), US@Fibers generated ROS under ultrasound irradiation. In the wet state, ROS diffused outward from the acoustic‐electric couplers, creating a clear zone of inhibition. The largest inhibition zone diameter was observed in the US@Fibers group, indicating the strongest antibacterial capability. To investigate the antibacterial efficacy of the acoustic‐electric couplers, we used SYTO9/PI staining to assess bacterial viability. SYTO9 marks bacteria with intact cell membranes, producing green fluorescence (indicating live cells), while PI marks bacteria with damaged cell membranes, producing red fluorescence. As shown in Figure 4F, Staphylococcus aureus and E. coli without ultrasonic irradiation exhibit strong green fluorescence. As a comparison, both US@Fibers and US@BaTiO_3_@Fe groups were more red fluorescent after ultrasound irradiation, indicating ROS production resulted in improved clearance efficiency for both bacterial species.

### 2.5 Dose‐Response Relationship of the Acoustic‐Electric Couplers: Transcriptomic Sequencing

**Figure 5:**
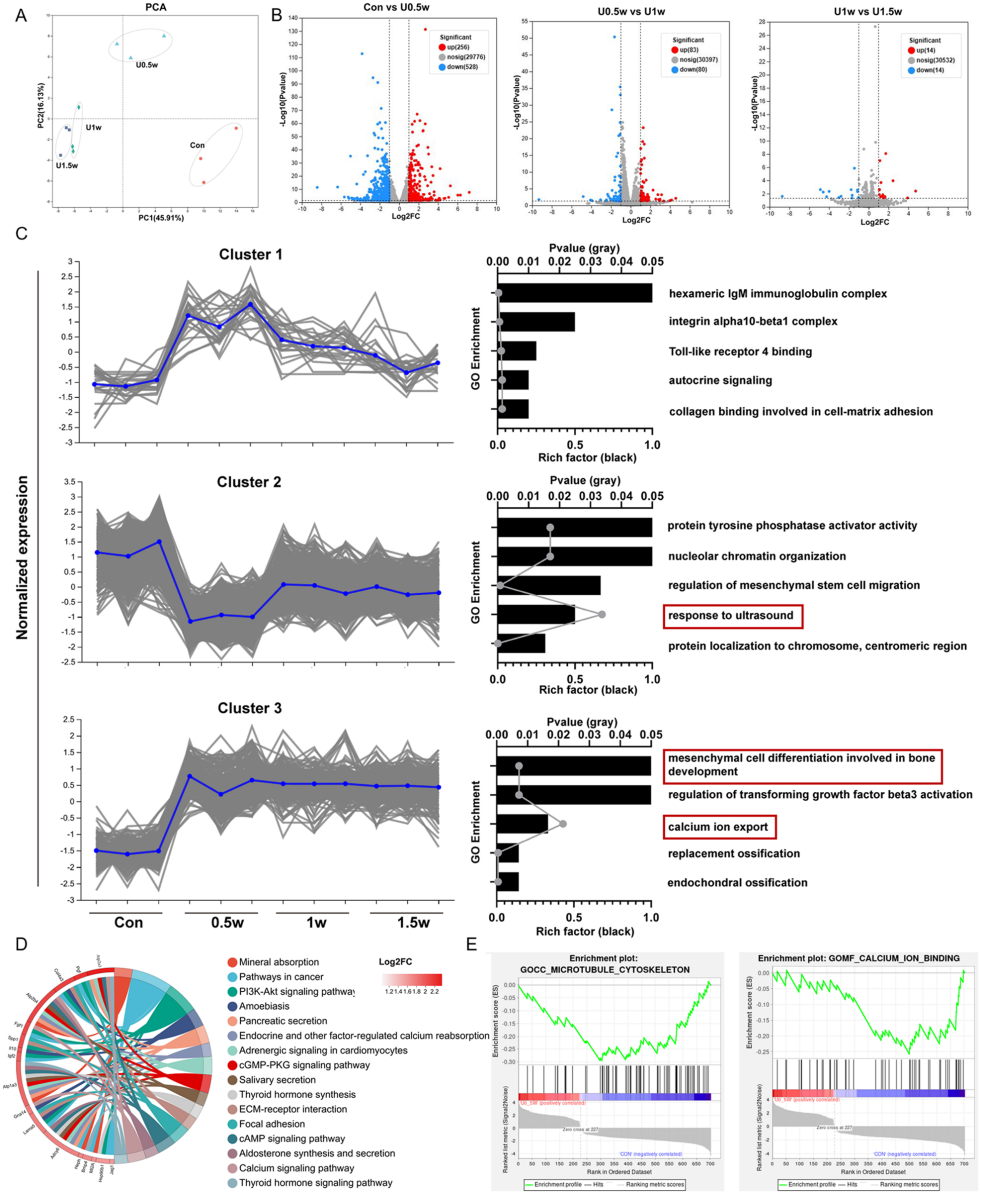
RNA Sequencing Analysis of the Dose‐Response Relationship of the Acoustic‐electric Couplers. A) Principal Component Analysis (PCA) among different groups. B) Volcano plot of DEGs from RNA‐seq data among groups. The X‐axis and Y‐axis represent log2 fold change and ‐log10 *p*‐value, respectively. Green dots indicate genes with log2 FoldChange > 1 and padj < 0.05. C) GO enrichment analysis of pathways associated with genes in three different clusters (log2 FoldChange > 1 and padj < 0.05). D) KEGG enrichment analysis of pathways associated with DEGs between the Control and 0.5 W groups. E) Representative pathways with significant differences from Gene Set Enrichment Analysis (GSEA).

The acoustic‐electric couplers can precisely generate electrical signals through mechanical activation by ultrasound.^[^ ^36^ ^]^ However, to effectively link the two critical stages of repair—antibacterial activity and osteogenesis—fine‐tuning of the repair process is essential. Clarifying the dose‐response relationship of the acoustic‐electric couplers is thus crucial. Utilizing transcriptomic sequencing to systematically analyze the relationship between ultrasound parameters (intensity, frequency, duration) and the stimulatory effects of electrical signals is an ideal approach. This method can elucidate the mechanisms by which ultrasound‐activated electrical signals regulate cell metabolism and osteogenesis‐related genes, while also identifying key regulatory factors in the biological signal transduction efficiency during the acoustic‐electric coupling process. To investigate the molecular mechanisms by which the acoustic‐electric couplers promote osteogenic differentiation of BMSCs, we conducted RNA sequencing in four groups: Control, 0.5 W, 1 W, and 1.5 W. Principal component analysis (PCA) and correlation heatmap results showed good distribution of relative expression abundance, with differences between groups but not within groups (**Figure** **5**A; Figure S8, Supporting Information). We then compared differentially expressed genes (DEGs) between Control versus 0.5 W, 0.5 W versus 1 W, and 1 W versus 1.5 W groups. The Control versus 0.5 W comparison had the most DEGs (784), indicating significant changes in gene expression due to ultrasound exposure. Between 0.5 and 1 W groups, there were 163 DEGs, with 83 upregulated and 80 downregulated genes (Figure 5B). Only 24 DEGs were found between the 1 and 1.5 W groups, suggesting limited efficiency in regulating the transcriptional process of BMSCs with excessively high‐intensity ultrasound. To further observe the transcriptional changes associated with varying ultrasound intensities, we selected three gene clusters based on transcriptional levels (Figure 5C). Cluster 1 contained genes with significantly higher transcriptional levels in the low‐intensity ultrasound group (0.5 W) compared to the other groups. Cluster 2 included genes with significantly lower transcriptional levels in the low‐intensity ultrasound group compared to the Control and high‐intensity ultrasound groups (1 and 1.5 W). Cluster 3 consisted of genes significantly upregulated after ultrasound stimulation. Through GO enrichment analysis, Cluster 1 was significantly enriched in pathways such as “integrin alpha10‐beta1 complex” and “collagen binding involved in cell‐matrix adhesion.” This suggests that low‐intensity ultrasound may trigger intracellular transcriptional activation by micro‐stimulating the cell membrane of BMSCs. Cluster 2 results indicated that DEGs were enriched in pathways such as “regulation of mesenchymal stem cell migration,” “response to ultrasound,” and “nucleolar chromatin organization,” suggesting that low‐intensity ultrasound is more effective in enabling BMSCs to respond to ultrasound signals. Finally, Cluster 3 results showed that ultrasound stimulation significantly enhanced pathways involved in “mesenchymal cell differentiation in bone development” and “endochondral ossification,” with calcium ion export being highly associated. Further analysis of KEGG enrichment of DEGs between the Control and 0.5 W groups revealed that calcium signaling pathways were significantly enriched (Figure 5D). Gene Set Enrichment Analysis (GSEA) results further confirmed the importance of calcium pathway alterations (Figure 5E) and indicated significant enrichment in the “Microtubule Cytoskeleton” pathway, supporting our hypothesis that the acoustic‐electric couplers can mediate biological signal transduction between cell nucleus and cell membrane, thus affecting transcription function. Transcriptomic sequencing results are expected to reveal the molecular‐level regulatory mechanisms of the acoustic‐electric couplers on cell functions, providing a theoretical basis for optimizing ultrasound activation parameters and material design (the selected ultrasound intensity for subsequent osteogenesis experiments was 0.5 W). These findings also highlight the need to further investigate the biological signal transduction process between the cell nucleus and cell membrane in response to ultrasound‐electrical stimulation, which is key to precisely regulating the continuous antibacterial‐osteogenic treatment.

### 2.6 Biocompatibility and Osteogenic Effects of the Acoustic‐Electric Couplers

**Figure 6:**
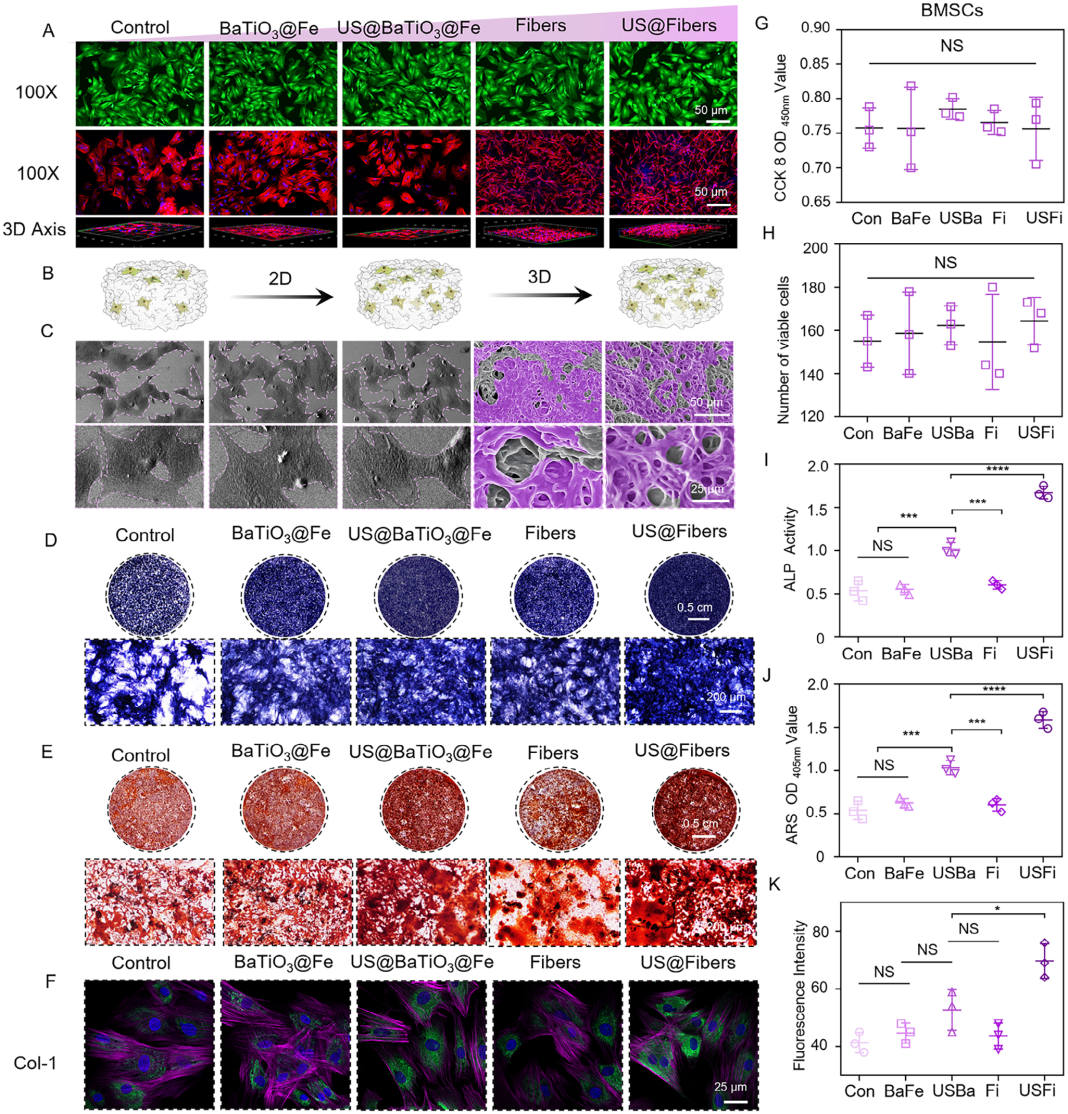
Effects of Acoustic‐electric Couplers on Osteogenic Differentiation of BMSCs. A) Live/dead staining and cytoskeleton staining images of BMSCs co‐cultured with short fiber scaffolds. B) Schematic illustration of cell growth patterns on acoustic‐electric couplers. C) SEM images of BMSCs in different groups. D) ALP staining of BMSCs. E) Alizarin Red staining results. F) IF images of Collagen I. G, H) CCK‐8 and live/dead staining statistical charts of acoustic‐electric couplers. I, J, K) Quantitative analyses of ALP staining, Alizarin Red staining, and immunofluorescence, respectively.

As an implantable cell scaffold, US@Fibers must first demonstrate excellent biocompatibility. BMSCs are important multipotent cells in bone tissue engineering. Due to their multilineage differentiation potential, self‐renewal capacity, immune regulatory functions, and other potential activities, BMSCs have become a crucial source of seed cells in regenerative medicine.^[^ ^37^, ^38^ ^]^ The composite fiber scaffold was thus exposed to live/dead staining, cytoskeleton staining, CCK‐8 assays, and cell surface morphology analysis after BMSCs were directly seeded onto the scaffold surface in this experiment. The experimental group of this part is as follows: Control, BaTiO_3_@Fe, US@BaTiO_3_@Fe, Fibers, and US@Fibers. First, live/dead staining was performed on BMSCs cultured on the composite hydrogel scaffolds for 4 days. As shown in **Figure** **6**A,H, only a few red fluorescence signals were observed, indicating cell death, while the remaining cells exhibited good morphology, viability, and normal proliferation rates without significant differences. Second, cells directly seeded on the fiber scaffolds and culture plates were subjected to cytoskeleton staining. After 4 days, cells showed good adhesion and extension, with visible filamentous protein structures in the cytoskeleton. Notably, cells on the fiber scaffolds extended more slowly than those on culture slides, likely due to fewer adhesion sites. Additionally, a 3D perspective revealed that BMSCs seeded on the scaffolds grew in a 3D manner, indicating excellent biocompatibility of the fibers and providing a foundation for subsequent repair stages (Figure 6B). Third, SEM was used to observe BMSCs seeded on the fiber scaffolds. As shown in Figure 6C, cells exhibited good extension and adhesion to the composite biomimetic fiber scaffolds or culture slides. Notably, cells on the composite fiber scaffolds grew along the porous structure, forming a porous cellular architecture that establishes a solid foundation for subsequent vascular ingrowth and nutrient supply. Fourthly, cell proliferation was assessed using the CCK‐8 on Day 4. Despite the moderate proliferation of cells on the scaffolds, there were no discernible group differences. This indicates that cell proliferation rates were not affected on the composite scaffolds, confirming their low cytotoxicity and suitability as biomaterials (Figure 6G). In summary, the composite fiber scaffolds promoted the proliferation of BMSCs. Moreover, ultrasound parameters did not significantly affect cell growth or proliferation rates. Reducing cytotoxicity and improving the adhesion and proliferation of multipotent stem cells are prerequisites for linking antibacterial and osteogenic processes and promoting rapid recovery in infectious bone repair. The toxicity of GO and Ba components: The long‐term toxicity of free‐state nanomaterials cannot be ignored in safety assessment. Studies have shown that GO and barium titanate‐based nanomaterials have low biological toxicity and good tissue tolerance when present in reasonable amounts and fixed on scaffolds.^[^ ^39^, ^40^ ^]^ In this study, both GO and barium titanate were firmly attached to the surface of 3D short fiber scaffolds through polydopamine to form stable composite coatings, rather than existing in a free state in the body. We demonstrated their stable existence through XPS, XRD, and SEM tests. Additionally, results from CCK‐8, live‐dead cell staining, and tissue sectioning showed that the GO and barium titanate‐coated scaffolds did not exhibit obvious toxic reactions, indicating their good biocompatibility.

Given the excellent biocompatibility of the acoustic‐electric couplers, their osteogenic potential was further validated. BMSCs are multipotent stem cells that can differentiate into osteoblasts under appropriate induction conditions.^[^ ^41^, ^42^ ^]^ This section was divided into three parts, with the following groups: Control, BaTiO_3_@Fe, US@BaTiO_3_@Fe, Fibers, and US@Fibers. BMSCs seeded on fiber scaffolds were induced with osteogenic medium and subjected to ALP staining and Alizarin Red staining at 7 and 21 days. Alkaline phosphatase (ALP) is an early marker in the osteogenic differentiation process, typically upregulated during the initial stages of BMSC differentiation into osteoblasts. Therefore, ALP staining can detect and confirm whether BMSCs have begun osteogenic differentiation. As shown in Figure 6D, the ALP staining intensity of US@Fibers group was significantly higher than that of other groups, and quantitative analysis also showed the same results (Figure 6I). Alizarin Red staining assesses whether BMSCs have successfully differentiated into osteoblasts and produced a mineralized matrix under osteogenic induction.^[^ ^43^ ^]^ Alizarin Red specifically binds to calcium salts (mainly hydroxyapatite), forming red precipitates. As shown in Figure 6E,J, the size and quantity of mineralized nodules in US@Fibers group were significantly higher than those in other groups. This indicates the highest calcium deposition efficiency in the US@Fibers group, confirmed by quantitative Alizarin Red S analysis. To further evaluate the osteogenic capacity of the acoustic‐electric couplers, IF assays were performed on cells on composite fibers at 7 days to detect the expression levels of proteins (osteogenic‐related) (Figure 6F,K). Collagen I is the primary organic component secreted by osteoblasts and significantly increases during BMSC differentiation into osteoblasts. Immunofluorescence detection at 7 days revealed that the COLLAGEN I protein fluorescence intensity in the US@Fibers group was 1.69, 1.56, 1.32, and 1.59 times higher than that in the Control, BaTiO_3_@Fe, Fibers, and US@BaTiO_3_@Fe groups, respectively. The fluorescence intensity is proportional to COLLAGEN I synthesis, with strong signals indicating that the acoustic‐electric couplers promote osteogenic differentiation. Similar observations were made in the immunofluorescence experiments for osteocalcin (OCN) (Figure S9, Supporting Information).

### 2.7 Osteogenic Mechanism of the Acoustic‐Electric Couplers

**Figure 7:**
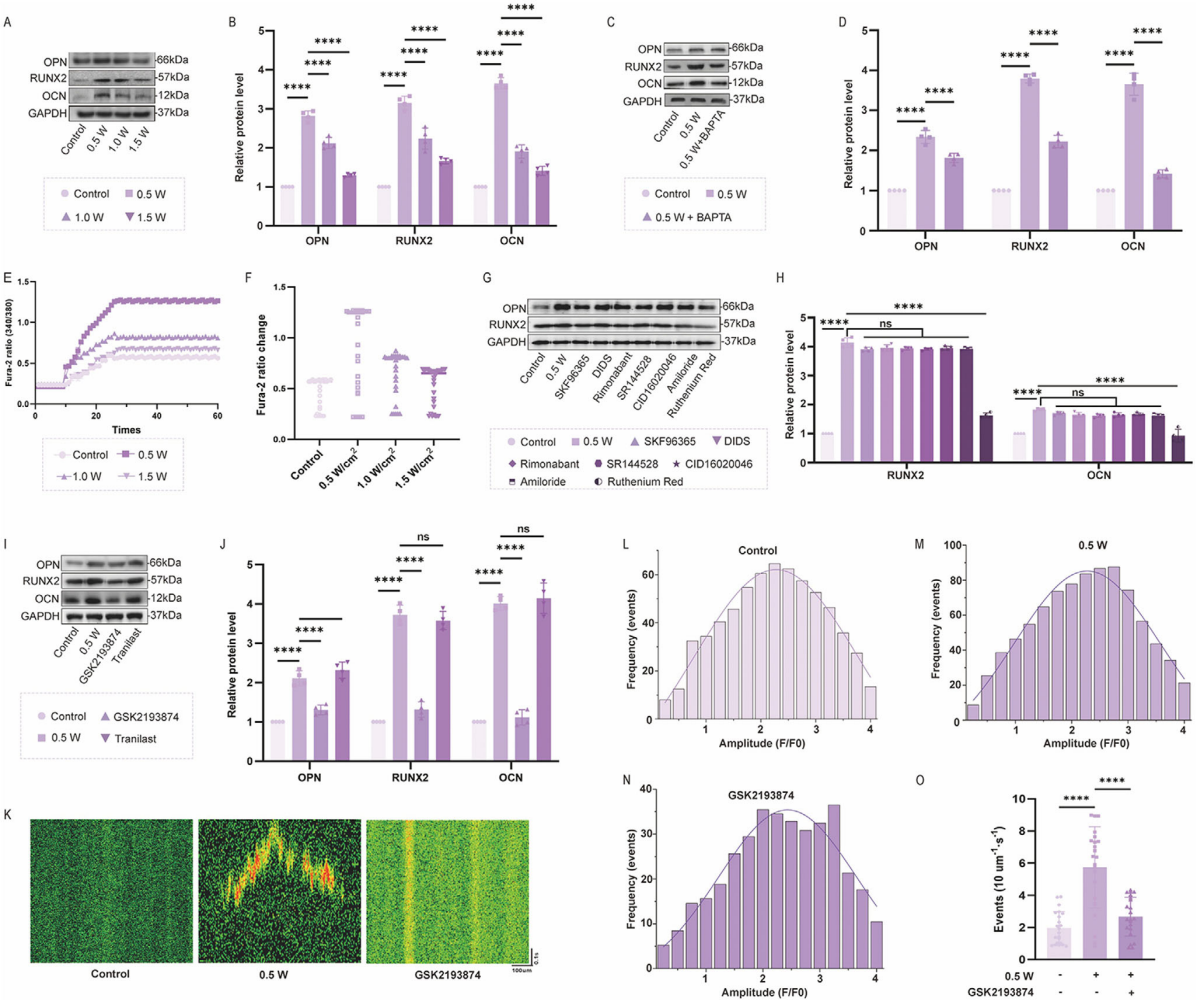
Exploration of the Osteogenic Mechanism of Acoustic‐electric Couplers A,B) Western blot analysis of osteogenic markers (OPN, RUNX2, OCN) under varying ultrasound intensities. The optimal osteogenic effect was achieved at 0.5 W, which was used for subsequent experiments. C,D) Western blot analysis demonstrating that the osteogenic effect induced by 0.5 W ultrasound was attenuated by BAPTA‐AM. E) Representative average trajectories of Fura‐2 Ca^2^⁺ imaging in osteoblasts at different ultrasound intensities. F) Scatter plot of Fura‐2 amplitude changes in osteoblasts. G,H) Western blot analysis of the impact of various ion channel inhibitors on osteogenesis induced by 0.5 W ultrasound. I,J) Effects of specific TRPV family ion channel blockers on osteogenesis induced by 0.5 W ultrasound. The TRPV4 antagonist GSK2193874 significantly reversed the osteogenic effect of ultrasound on BMSCs. K) Representative fluorescence surface plots of spontaneous Ca^2^⁺ sparks in osteoblasts under different treatments. GSK2193874 significantly reduced ultrasound‐induced calcium influx. L–N) Histogram analysis of Ca^2^⁺ spark frequency in osteoblasts under different treatments. O) Scatter plot of Ca^2^⁺ spark frequency.

**Figure 8:**
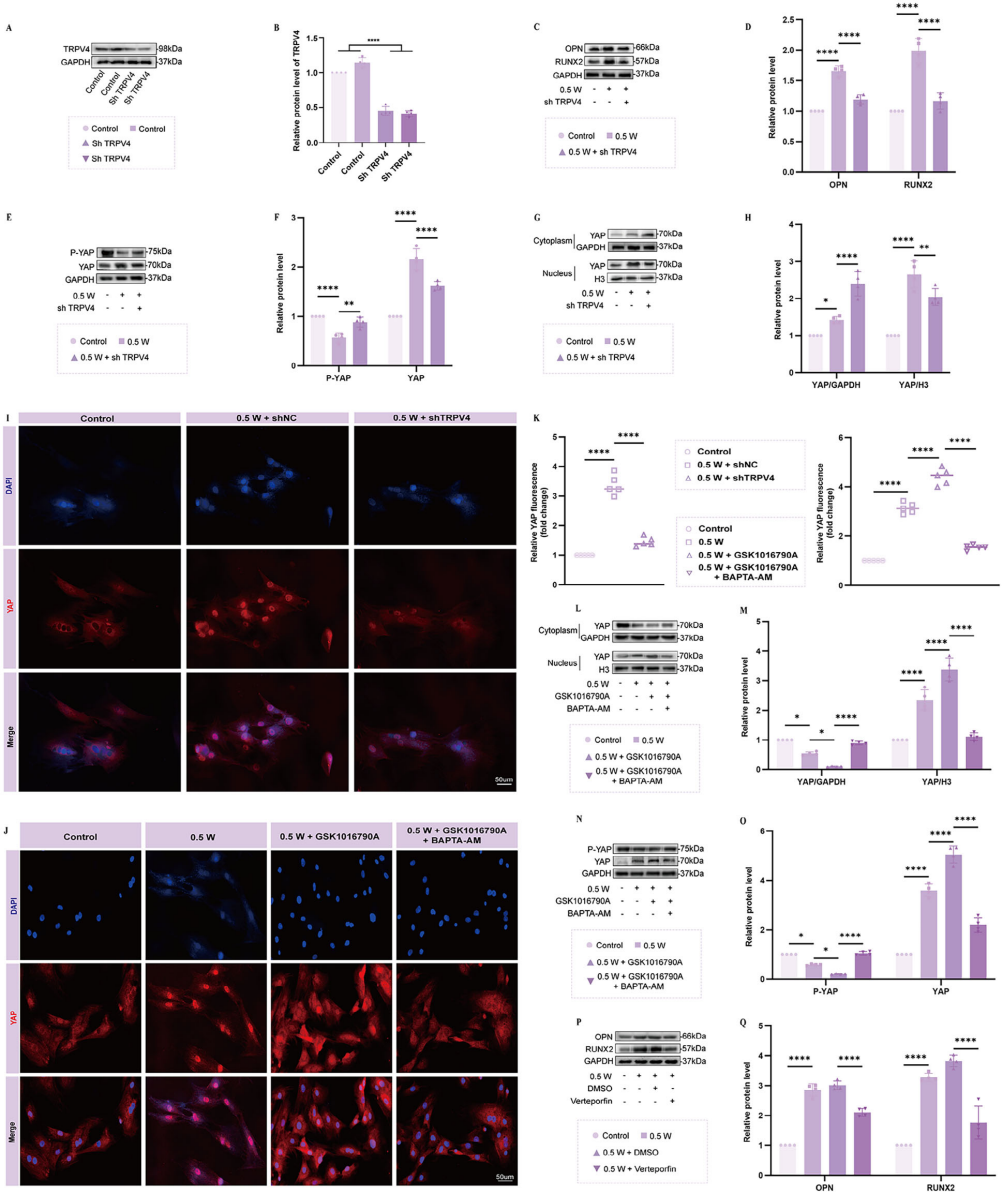
Exploration of the Osteogenic Mechanism of Acoustic‐electric Couplers A,B) Western blot analysis confirming successful TRPV4 knockdown using sh‐TRPV4. C,D) Western blot analysis of osteogenic markers showing that TRPV4 knockdown reversed the osteogenic effect induced by ultrasound. E,F) Expression levels of YAP and p‐YAP in osteoblasts under different treatments. G,H) TRPV4 knockdown reversed the ultrasound‐induced nuclear translocation of YAP. I, K) Fluorescence images showing that ultrasound promotes YAP nuclear translocation, which is blocked by TRPV4 knockdown. J, K) Fluorescence images demonstrating that the TRPV4 agonist GSK1016790A enhances ultrasound‐induced YAP nuclear translocation, while calcium ion antagonists reverse this effect. L–M) Western blot analysis showing that the effect of GSK1016790A on YAP nuclear translocation under ultrasound is reversed by calcium ion antagonists. N,O) Wb analysis showing that the inhibitory effect of GSK1016790A on YAP phosphorylation under ultrasound is reversed by calcium ion antagonists. P,Q) Western blot analysis of osteogenic markers showing that the osteogenic effect of ultrasound is reversed by the YAP inhibitor verteporfin.

**Scheme 2:**
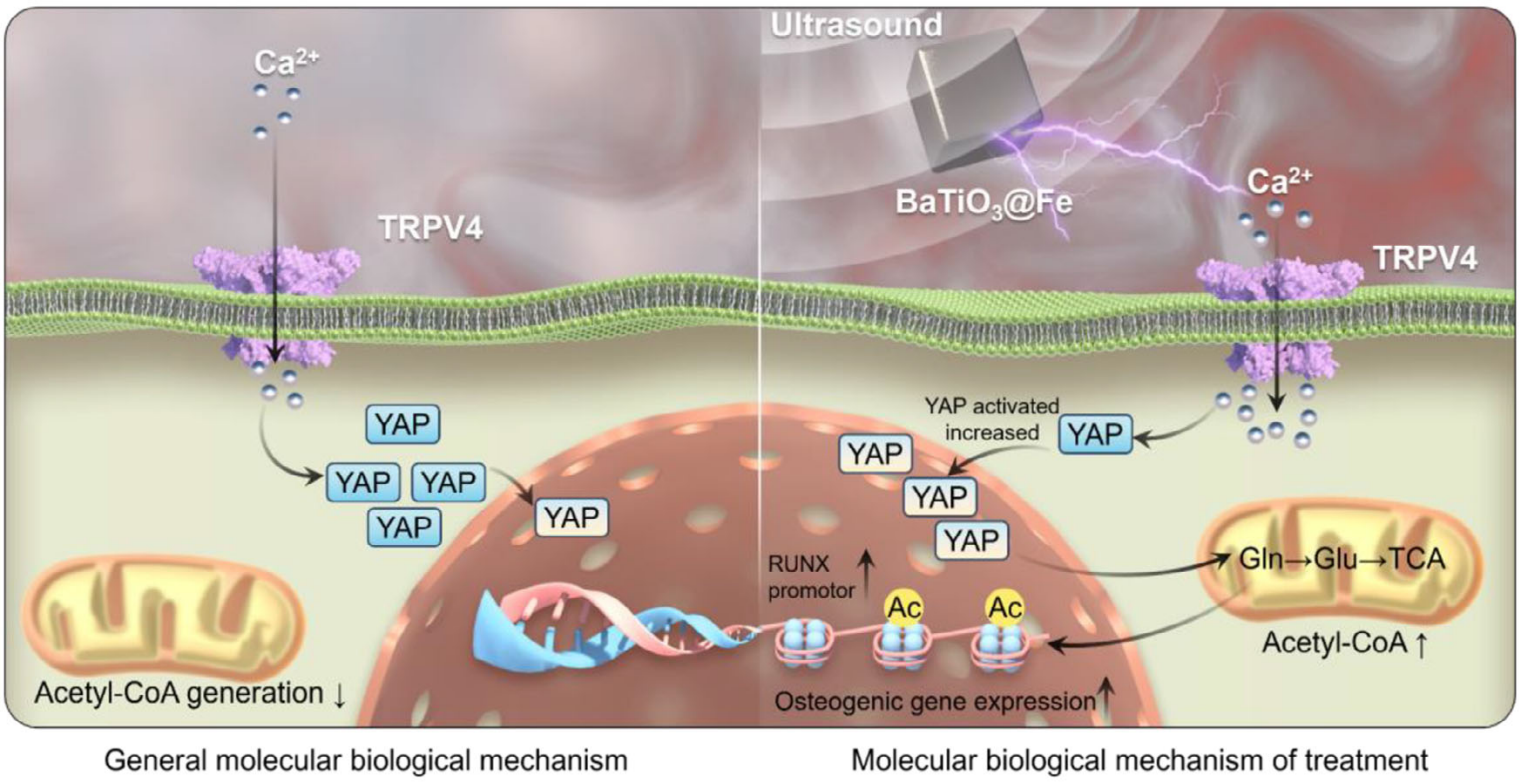
Mechanistic Illustration of Osteogenesis by Acoustic‐electric Couplers.

To further elucidate the osteogenic effects of the acoustic‐electric couplers at an ultrasound intensity of 0.5 W, we first assessed the expression of osteogenic markers OCN, OPN, and RUNX2 using Western blot. As shown in **Figure** **7**A,B, the osteogenic effect induced by the acoustic‐electric couplers was most pronounced at an ultrasound intensity of 0.5 W. Notably, pre‐treatment with the extracellular calcium chelator BAPTA significantly attenuated the osteogenic effect when followed by ultrasound stimulation (Figure 7C,D). Since the release of Ca^2^⁺ from intracellular stores and the influx of extracellular Ca^2^⁺ are two potential sources of increased intracellular free Ca^2^⁺, we monitored calcium flux using the Fura‐2 AM probe. We found that ultrasound induced calcium influx, which peaked at 0.5 W (Figure 7E,F). This suggests that the increase in intracellular free Ca^2^⁺ was primarily due to the absorption of extracellular Ca^2^⁺ rather than the release from endogenous Ca^2^⁺ stores. Additionally, real‐time calcium signaling in BMSCs was observed using a live‐cell imaging system. Fluorescence imaging of calcium probes showed that both the amplitude and frequency of calcium oscillations increased significantly under ultrasound stimulation, peaking at an ultrasound intensity of 0.5 W (Movies S1–S4, Supporting Information). The experimental data suggest that the extracellular calcium flux triggered by external stimuli could be a key factor in facilitating the ultrasound‐induced osteogenic differentiation of BMSCs. Our subsequent experimental protocol involved pre‐treating BMSCs with an assortment of calcium channel inhibitors targeting TRPV, TRPC, TRPM, CBARP/VGCC channels, and blockers of G protein‐coupled receptors (GPCRs), specifically CNR1/CB1, CNR2/CB2, and GPR55. From the observations, antagonists for GPR55, CB1, CB2, VDAC1 inhibitor, TRPC blocker, and T‐type calcium channel blocker did not influence ultrasound‐induced osteogenesis. However, RuR, the TRPV channel blocker, remarkably counteracted the osteogenic differentiation of BMSCs induced by ultrasound. (Figure 7G,H). To further investigate the specific ion channels involved in this process, we employed specific blockers targeting each ion channel within the TRPV family. It was revealed that the TRPV4 antagonist GSK2193874 could reverse the osteogenic effect of ultrasound on BMSCs, whereas inhibitors of TRPV1 or TRPV2 (tranilast) were ineffective in this regard. (Figure 7I,J). Consistent with these findings, monitoring calcium flux using the Fluo‐4AM probe revealed that GSK2193874 significantly reduced ultrasound‐induced calcium influx (Figure 7K–O). Subsequently, we used shRNA targeting TRPV4 to investigate its role in ultrasound‐induced osteogenesis. As shown in **Figure** **8**A,B, TRPV4 was successfully knocked down. We found that interfering with TRPV4 partially reversed the osteogenic effect induced by ultrasound (Figure 8C,D). The data imply that TRPV4 forms an essential component in the process of ultrasound‐induced osteogenesis. YAP, which functions as a mechanosensitive transcriptional activator, exerts significant influence on cell differentiation and is responsive to mechanical stress.^[^ ^44^ ^]^ Since ultrasound‐induced mechanical stress activates TRPV4,^[^ ^45^ ^]^ we hypothesized that ultrasound promotes osteogenic differentiation of BMSCs through TRPV4‐mediated activation of YAP. In our initial investigation, we assessed YAP expression and phosphorylation in response to ultrasound stimulation. The data revealed an upregulation of YAP expression coupled with a marked reduction in its phosphorylation. However, when TRPV4 was knocked down, these effects were attenuated (Figure 8E,F). In addition, immunofluorescence (IF) was carried out to explore YAP subcellular localization. It was observed that ultrasound triggered the nuclear translocation of YAP, and this phenomenon was hindered when TRPV4 was knocked down. (Figure 8I–K). Consistent results were obtained from nuclear‐cytoplasmic fractionation analysis (Figure 8G,H). Additionally, since calcium ions act as second messengers involved in action potential regulation and transcription,^[^ ^46^ ^]^ we speculated that Ca^2^⁺ is essential for ultrasound‐induced TRPV4‐YAP mechano‐transduction. To explore the function of calcium influx, in vitro studies were carried out. It was found that GSK1016790A, a TRPV4 agonist, could boost the ultrasound‐induced nuclear translocation of YAP, augment its expression, and at the same time, inhibit YAP phosphorylation. (Figure 8J,N,O). The reversal of these effects by pre‐incubation with BAPTA‐AM corroborates the essential role of intracellular calcium accumulation in the ultrasound – induced activation of the TRPV4 – YAP pathway. (Figure 8L,M). To further explore the role of YAP in ultrasound‐induced osteogenesis, we pre‐treated BMSCs with the specific YAP inhibitor verteporfin before ultrasound stimulation and assessed osteogenic protein expression using Western blot. As expected, inhibiting YAP in BMSCs attenuated the ultrasound‐induced osteogenic response (Figure 8P,Q). In summary, these data demonstrate that ultrasound promotes osteogenic differentiation of BMSCs through the TRPV4/Ca^2^⁺/YAP axis (**Scheme** **2**).

### 2.8 In Vivo Anti‐Infection and Osteogenic Effects of the Acoustic‐Electric Couplers

It is well‐known that infection in bone defects can lead to local inflammatory responses, bone destruction, and necrosis, which inhibit the function of osteoblasts and impede new bone formation.^[^ ^47^ ^]^ Uncontrolled infections can exacerbate local tissue damage, leading to severe complications such as osteomyelitis and even bone necrosis.^[^ ^48^ ^]^ Therefore, effectively controlling infections at an early stage is a prerequisite for successful bone repair, and integrating infection control with subsequent osteogenesis is crucial for therapeutic success. The presence of infection not only delays bone healing but can also cause further damage and functional loss in bone tissue. To comprehensively evaluate the anti‐infection and osteogenic effects of the acoustic‐electric couplers, an infectious segmental bone defect model in the rat femur (3 mm in diameter) was employed. The groups were divided as follows: Control, US@BaTiO_3_@Fe, Fibers, and US@Fibers. Evaluations included osteogenesis and anti‐infection assessments, with histology, in vivo fluorescence imaging, bacterial standard plate counting, and 3D micro‐CT reconstruction used to assess the quantity and morphology of regenerated bone tissue and the antibacterial efficacy within the defects.

**Figure 9:**
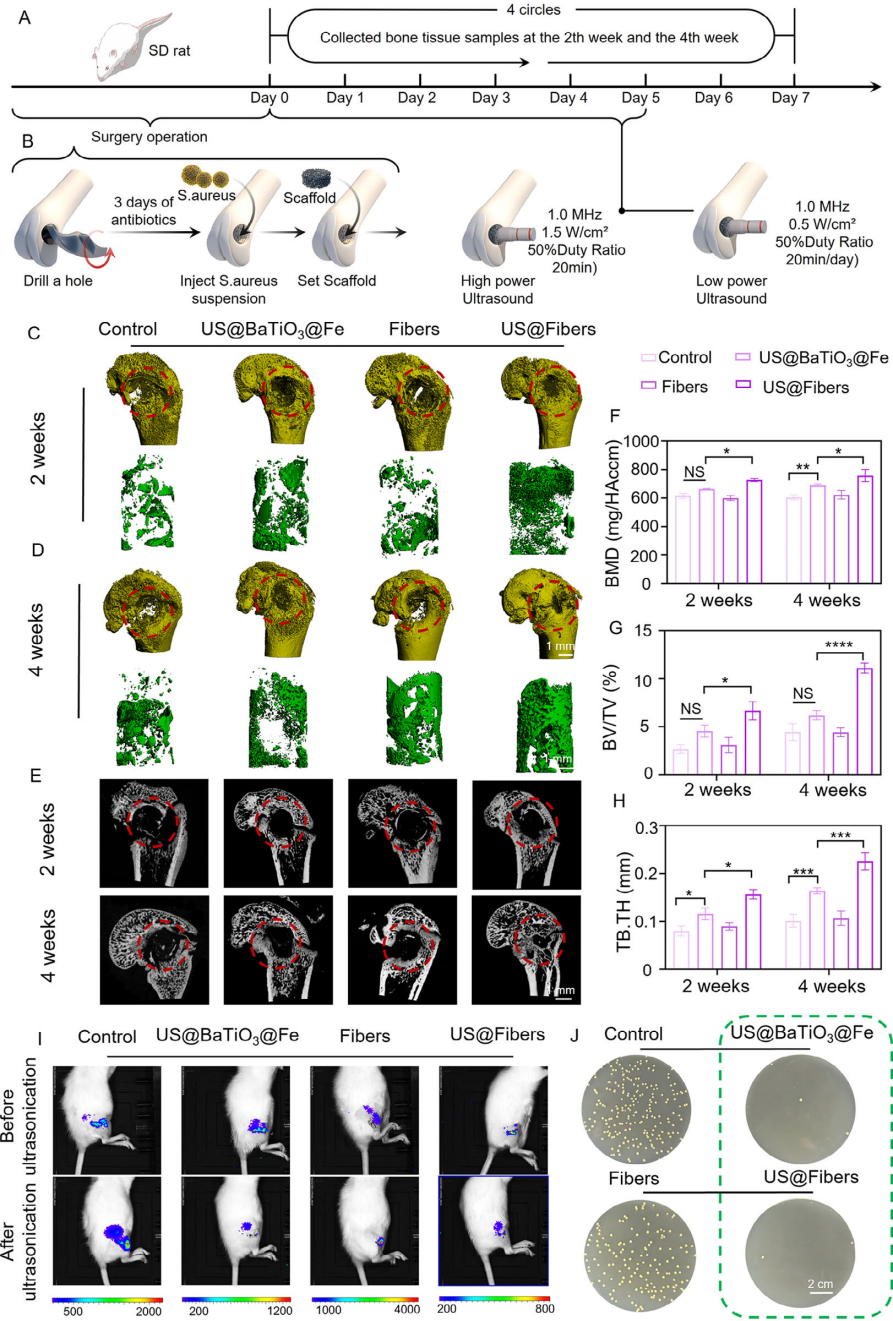
In Vivo Osteogenic and Antibacterial Therapeutic Effects of Acoustic‐electric Couplers A) Schematic diagram of the animal surgical procedure. B) Schematic illustration of the osteogenic mechanism of acoustic‐electric couplers. C, D) 3D micro‐CT reconstruction images at 2 and 4 weeks. E) Tomographic images from micro‐CT. F–H) Bone morphometric analysis of rat femoral defects. Total callus volume, BMD, BV/TV, and Tb.Th. I, J) In vivo imaging of acoustic‐electric couplers in animals and bacterial standard plate counting.

All experimental procedures were carried out on male Sprague–Dawley rats aged 10–12 weeks. We performed high–resolution micro – CT scans and 3D reconstructions of femoral defects at 2 and 4 weeks after the surgical procedure. (**Figure** **9**A,B). As shown in Figure 9C,D,E, no significant bone regeneration was observed in the blank control and pure fiber groups, with clear fracture lines visible at the defect sites. This may be due to the implantation of Staphylococcus aureus, whose toxins and metabolic byproducts could stimulate local osteoclast activity, increasing bone resorption and exacerbating bone loss in the defect area. Notably, in the Fibers group, although fibers have been proven to have a positive effect on bone repair as a filling material for bone defects, the inability to effectively control infection led to the fibers acting as a “nursery” for bacterial colonization and infection, providing a favorable environment for bacterial growth. Thus, similar to the blank control group, effective bone formation was not achieved. In contrast, the US@BaTiO_3_@Fe and US@Fibers groups showed varying degrees of bone regeneration. Particularly at 4 weeks, the US@Fibers group exhibited new bone tissue that almost filled the majority of the defect area. Compared to the US@BaTiO_3_@Fe group, both groups possessed acoustic‐electric coupling capabilities. However, the presence of the fiber scaffold in the US@Fibers group integrated “power generation” and “conductivity,” mimicking the microstructure of natural bone tissue, including porosity, pore size distribution, and surface properties, thereby better promoting the attachment, proliferation, and differentiation of osteoblasts. This provided a significant advantage for bone regeneration. In contrast, pure nanoparticles often fail to provide sufficient mechanical support, leading to potential instability under load and limiting their application in bone repair. To more precisely observe the defect area and further quantify new bone mass, micro‐CT reconstruction technology was used to visualize the entire femur and the internal structure of the defect. Over time, new bone formation was observed from the defect margins toward the center in all experimental groups. Additionally, the US@Fibers group showed superior defect regeneration compared to the other groups. This phenomenon indicates that the acoustic‐electric couplers effectively converted external ultrasound mechanical energy into electrical signals through the acoustic‐electric couplers. With the early effective antibacterial activity as the foundation, it modulated multi–level bioelectrical signals in vivo and enhanced the repair of infectious bone defects. Of note, analyses of BMD, Tb.Th, Tb.Sp, and BV/TV showed that the experimental groups presented significantly higher BMD, Tb.Th, and BV/TV than the other groups, but lower Tb.Sp (Figure 9F,G,H; Figure S10, Supporting Information). This suggests that the US@Fibers group effectively linked the antibacterial and osteogenic processes, rapidly promoting the repair of infectious bone defects. Furthermore, the acoustic‐electric couplers can generate reactive oxygen species (ROS) at an ultrasound intensity of 1.5 W cm^−^^2^, effectively killing invading Staphylococcus aureus. As shown in Figure 9I,J; Figure S11 (Supporting Information), under ultrasound irradiation, bacterial growth was effectively inhibited, both in in vivo imaging of small animals and in bacterial standard plate counting after tissue collection. This laid a solid foundation for subsequent osteogenesis.

### 2.9 Histological Analysis of In Vivo Anti‐Infection and Osteogenesis by the Acoustic‐Electric Couplers

**Figure 10:**
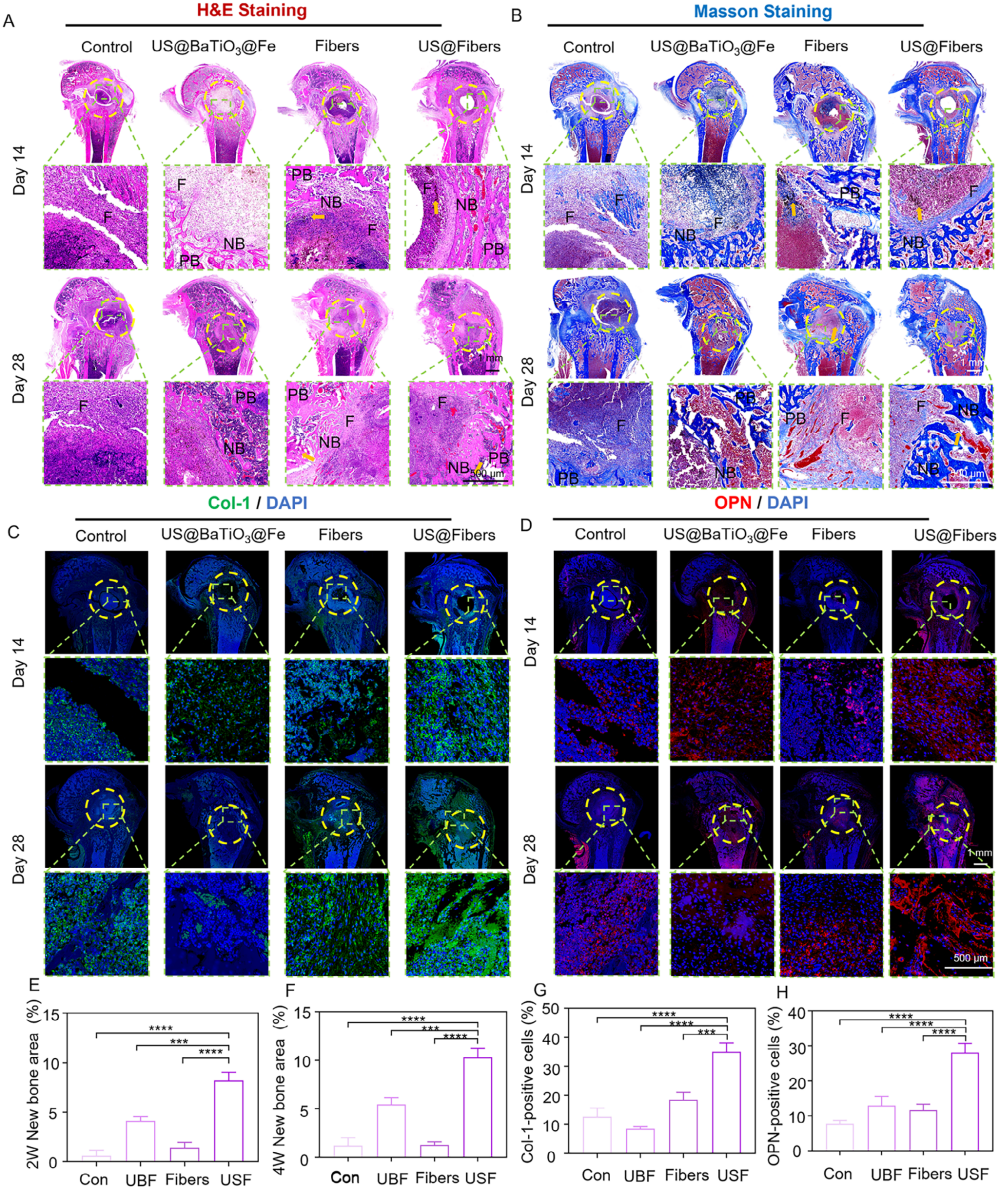
Histological Staining and Scoring to Assess In Vivo Therapeutic Effects in Femoral Defects A) H&E staining. B) Masson staining. Yellow arrows denote BaTiO_3_ nanoparticles. C) Immunofluorescence staining for type I collagen. D) Immunofluorescence staining for osteopontin (OPN). E, F) Quantitative assessment of osteogenic area. G, H) Quantitative analysis of immunofluorescence for Collagen I and OPN.

To thoroughly evaluate the osteogenic capability of the acoustic–electric couplers, histological evaluation of new bone regeneration in femoral defects at 2 and 4 weeks post–surgery was carried out using hematoxylin and eosin (H&E) and Masson staining. NB stands for newly formed bone tissue, PB for pre–existing bone tissue, and F for fibrous tissue. Additionally, the defect area is outlined with a yellow dashed line for clarity, and the fiber scaffold is marked with a yellow arrow. As depicted in **Figure** **10**A,B,E, F, the composite fiber scaffold exhibited a certain degree of degradation at both 2 and 4 weeks after surgery. The black parts indicated by the yellow arrows are barium titanate nanoparticles, which prove that, within the observed experimental period, barium titanate has good biological safety and does not interfere with bone formation. In the blank control group, the infiltration of Staphylococcus aureus disrupted the bone marrow microenvironment within the defect area, thereby hindering the osteogenic process. Excessive aggregation of inflammatory cells (such as macrophages and leukocytes) and the secretion of cytokines impacted bone regeneration and repair. It was observed that, up to 4 weeks, only minimal new bone formation occurred, and in some cases, no new bone was formed. The regenerated bone tissue was insufficient to restore the original mechanical structure, thereby increasing the risk of refracture. A similar phenomenon was observed in the Fibers group. Although the implanted scaffold provided the necessary filling material for bone defect repair, bacterial invasion disrupted the local immune microenvironment, leading to repair failure. In contrast, the US@BaTiO_3_@Fe and US@Fibers groups, which featured ultrasound‐mediated anti‐infection capabilities, effectively controlled the excessive immune destruction caused by bacterial invasion. This was attributed to the deformation of piezoelectric nanoparticles under ultrasound stimulation, generating an internal electric field that promotes bacterial cell membrane polarization and subsequently enhances the production of ROS. ROS oxidize the phospholipid layer of the cell membrane, increasing membrane permeability and causing structural damage and death of bacteria. Notably, the US@Fibers group exhibited superior osteogenic effects compared to the US@BaTiO_3_@Fe group. This advantage can be attributed to two main factors. First, while effectively controlling infection through “power generation,” the “conductivity” effect of the US@Fibers group continued to recruit surrounding electric fields, enhancing the activity of BMSCs and promoting osteogenesis. Second, the biomimetic fiber scaffold, as an essential implant material for bone defect repair, provided a favorable growth environment for BMSCs. Unlike pure nanoparticles, which are easily cleared by the immune system, the fiber scaffold demonstrated better osteogenic outcomes.

To further evaluate the osteogenic effects, immunofluorescence analysis was performed on tissue sections, with Collagen I and OPN as markers. As shown in Figure 10C,D,G, H, the US@Fibers group exhibited higher mean fluorescence intensity and larger fluorescence areas compared to the Control, US@BaTiO_3_@Fe, and Fibers groups, consistent with the results from H&E and Masson's trichrome staining. Immunofluorescence labeling of collagen type I is an important indicator of osteoblast differentiation and bone matrix synthesis. Osteopontin (OPN) plays a crucial role in fracture healing and bone repair by modulating local immune responses and promoting the migration and proliferation of osteoblasts, thereby facilitating bone tissue repair.^[^ ^49^ ^]^ OPN acts as a key molecule in the fracture healing process, promoting inflammatory responses in the wound area and stimulating the proliferation and differentiation of osteoblasts.^[^ ^50^ ^]^ In summary, in the repair of rat infectious bone defects, ultrasound at 1.5 W cm^−^^2^ effectively controlled bacterial invasion at the infection site, while ultrasound at 0.5 W cm^−^^2^ continuously regulated the subsequent osteogenic process. This approach achieved the regulation of multi‐level bioelectrical signals in vivo, integrating “power generation” and “conductivity” in an orderly manner to accelerate the repair of infectious bone defects. This sequential experimental design (first conducting antibacterial treatment, then promoting bone formation) is in line with the pathological process of infectious bone defects: Only after eliminating the pathogenic bacteria can the inflammatory microenvironment be relieved, thereby creating favorable conditions for bone formation and differentiation.^[^ ^51^ ^]^ Moreover, the continuous ultrasonic stimulation in the two stages not only activated the antibacterial effect of the fibers through acoustic‐electrical coupling in the early stage, but also promoted the differentiation of osteoblasts by regulating the intracellular calcium ion concentration in the later stage, verifying the synergistic effect of the sequential strategy and the material's electro‐sensitivity. For acoustic‐electric coupling devices, future miniaturization and portable designs are necessary.^[^ ^52^ ^]^ During the operation, this device can be handheld by aligning it with the defect site through real‐time imaging (such as ultrasound guidance) to ensure accurate ultrasound stimulation during the fiber implantation process. After the operation, patients can use the wearable version (fixed by medical bandages) for daily stimulation, so that clinicians can adjust the parameters remotely to reduce the need for frequent hospitalizations. Additionally, the operation of this device can be combined with the existing orthopedic surgical procedures without the need for specialized training for surgeons, thereby further reducing the barriers to clinical implementation.

Although this research has achieved encouraging results, there are still some limitations that need to be acknowledged. First, the current animal models differ in complexity from human infectious bone defects: human defects usually involve larger volumes, longer infection durations, and factors that accompany other diseases that change the local microenvironment, all of which may affect the antibacterial and osteogenic effects of the materials in the clinical environment. To address these limitations and advance the technology, future research will focus on the following three aspects: (1) verification in large animal models to more accurately simulate the scale and microenvironment of human defects; (2) conducting long‐term safety assessments; (3) optimizing the portable acoustic‐electric devices.

## 3 Conclusion

In this study, we analysed the limitations of clinical electrical stimulation therapy. For the first time, we developed electrically sensitive heterogeneous short fibers through acousto‐electric coupling, which effectively promoted the repair of infected bone defects. Using an ion‐doping modification technique, we prepared iron‐doped barium titanate nanoparticles (BaTiO_3_@Fe). Through *π*–*π* conjugation interactions and the bio‐inspired redox properties of polydopamine, along with the multi‐site anchoring function of its catechol groups, we achieved the in situ deposition and reduction of graphene oxide and BaTiO_3_@Fe within a 3D short fiber matrix. This process created a composite acousto‐electric coupled material (acousto‐electric coupler) with both piezoelectric and conductive properties. The system, leveraging its synergistic piezoelectric‐conductive dual functionality, responds precisely to ultrasound field modulation. At an ultrasound intensity of 1.5 W cm^−^^2^, the material's piezoelectric‐conductive coupling effect specifically activates bacterial peroxisome pathways and necroptosis signalling, significantly inducing pathogen apoptosis. In contrast, at a lower ultrasound intensity of 0.5 W cm^−^^2^, the system regulates the TRPV4/Ca^2^⁺/YAP signalling axis, initiating a cascade that activates osteogenic differentiation in BMSCs (Schemes 1 and 2). This dual‐modal dynamic response mechanism enables cross‐scale transmission and optimized efficiency of bioelectrical signals. By employing spatiotemporal differential electrical regulation strategies, the system triggers a cascaded antibacterial‐osteogenic effect, effectively restoring the electro‐microenvironmental homeostasis of bone tissue and accelerating the repair process of infectious bone defects. The ultrasound‐responsive smart fiber material developed in this study offers an innovative solution for the precise electrical regulation of infected bone defects and holds significant potential for clinical translation.

## 4 Experimental Section

### Clinical Electrical Stimulation and Finite Element Analysis

Study Design and Ethical Approval: This single‐center, retrospective cohort study was approved by the institutional Ethics Committee, and all patients provided written informed consent. Medical records of adult patients (≥18 years) with radiographically and clinically confirmed bone fractures treated between May 2022 and May 2024 were reviewed, following the STROBE guidelines for observational research. Patient Selection: Inclusion criteria: 1) Adult patients (≥18 years) with closed bone fractures, and 2) completion of a standardized rehabilitation program. Exclusion criteria: 1) Open fractures (Gustilo‐Anderson IIIB/C), 2) Polytrauma (Injury Severity Score ≥16), 3) Preexisting neuromuscular or mobility‐limiting disorders, and 4) Incomplete electronic health records. Patients were categorized into two groups. 1) EST group (n = 88): received standard rehabilitation plus EST therapy. 2) No‐EST group (n = 27): received standard rehabilitation alone. Data Collection: Collected baseline data included age, sex, time from injury to treatment, fracture site (upper vs lower limb), surgical fixation, intramedullary nail usage, and concomitant osteoarthritis. Outcome measures, including functional assessments, inflammation markers, and radiographic healing, were systematically evaluated at baseline and post‐rehabilitation timepoints. Functional assessments incorporated the Visual Analog Scale (VAS) for pain intensity, Manual Muscle Strength Grading (MSG) for muscle strength, and Activities of Daily Living (ADL) scores for functional independence. Inflammation markers include white blood cell count (WBC), neutrophil percentage (NEUT%), and C‐reactive protein (CRP). Meanwhile, Fracture healing was also assessed radiologically by plain radiographs. Fracture healing was quantified by using a 4‐point Fracture Healing Score (FHS): 1 (absence of callus), 2 (visible callus), 3 (bridging callus), and 4 (fracture line obliteration). Based on the CT results of the patients, the finite element analysis of clinical electrical stimulation and ultrasound was carried out using COMSOL Multiphysics. The ethical number was 2025‐608‐01.

### Preparation and Characterization of Fe‐Doped Piezoelectric Nanoparticles

The tetragonal‐phase Fe‐doped barium titanate piezoelectric nanoparticles were synthesized via a hydrothermal method.^[^ ^53^ ^]^ Initially, 1.9 g of barium nitrate was dissolved in 50 mL of deionized water under continuous stirring until complete dissolution. Subsequently, 2.5 mL of titanium butoxide was added dropwise and stirred until homogenized. Next, 0.235 g of ferric chloride (Fe:Ba = 0.2) was introduced, followed by ultrasonic dispersion and stirring. Weighing 20 g of sodium hydroxide, and stirred it until entirely dissolved. Transferring the mixture into a hydrothermal reactor, and kept it at 200° C for 24 h. Once cooled, the powder obtained was washed to neutrality with 0.1 m hydrochloric acid, anhydrous ethanol, and deionized water, followed by drying at 60° C to get the final product. The nanoparticles' composition, crystal structure, morphology, and particle size distribution were analyzed via XRD, SEM‐EDS, TEM, and a nanoparticle size analyzer. Moreover, their piezoelectric properties, including single–point spectral measurements, were evaluated using PFM. For PFM analysis, the nanoparticles were evenly applied onto a 1 × 1 cm^2^ ITO conductive glass substrate as the bottom electrode. The piezoelectric response curves were then acquired in DART‐PFM mode.

### Preparation of the Acoustic‐Electric Couplers

To create the electrospun fibers, a gelatin/PLA composite (w/w = 80:20) was processed into membrane form following the method reported by the research group.^[^ ^54^ ^]^ The fiber membranes were cut into small pieces, added to t – butanol, and homogenized with an IKA T – 18 homogenizer (10 000 rpm) for 30 min. The fiber dispersion was then placed into a 24–well culture plate and lyophilized for 48 h. The crosslinking process took place at 180° C for 2 h, resulting in a stable electrospun fiber sponge (NS). The NS was immersed in a 4 mg mL^−1^ GO solution for 12 h, after which it was washed with deionized water to remove unbound GO. It was then placed in a dopamine (DA, 2 mg mL^−1^) solution containing BaTiO_3_@Fe (5 mg mL^−1^) at 60 °C for 24 h. This process yielded a biomimetic short–fiber scaffold with both “power–generating” and “conductive” properties. The scaffold's morphology was examined using a digital camera (Canon) and SEM (FEI), and the average fiber diameter was measured with ImageJ. The successful synthesis of the acoustic–electric couplers was confirmed by XRD, XPS (Kratos Analytical, UK), and FTIR.

### Characterization of the Acoustic‐Electric Couplers

This section comprehensively evaluates the mechanical properties, hydrophilicity, conductivity, and ultrasound responsiveness of the acoustic‐electric couplers. First, the mechanical performance of the scaffolds was assessed using a material testing machine (HY‐940FS, China) through cyclic compression tests. Next, the hydrophilicity of the acoustic‐electric couplers was tested using a contact angle experiment. Briefly, different fiber scaffolds were selected as test samples, ensuring that the surfaces were clean and free of contamination. A small droplet of test liquid was placed on the sample surface using a micro‐syringe, and the contact between the droplet and the solid surface was captured using a high‐definition camera or microscope. The contact angle was then measured using image analysis software. The conductivity of the scaffolds was evaluated according to the literature.^[^ ^54^ ^]^ The fiber scaffolds were placed in 50 mL centrifuge tubes and agitated at 37° C for 1, 5, 10, 15, and 20 days. After washing, the scaffolds were measured for resistivity using a resistivity meter (ST2742B, China). Under ultrasound irradiation, the short‐circuit voltage and open‐circuit current of the hydrogel were detected using a digital oscilloscope. The total ROS, •OH, and ^1^O_2_ were measured using a fluorescence spectrophotometer. After ultrasound treatment, rhodamine B (10 mg L^−1^), methylene blue (5 mg L^−1^), and TMB colorimetric assays (5 mg L^−1^) were used to react for a certain period. Centrifugation was performed to obtain the supernatant, following which the absorbance was determined. The generation of •OH, •O_2_^−^, and ^1^O_2_ under different ultrasound powers was quantitatively detected using electron spin resonance (ESR, Germany) with DMPO, CYPMPO, and TEMP as spin traps. The ultrasonic parameters were: intensity gradient of 1.5, 1, 0.5, 0W; duty cycle of 50%; frequency of 1 mHz.

### Cell Culture and RNA Sequencing

For the isolation of BMSCs, 4‐week‐old male SD rats (sourced from the Animal Experiment Center of Chongqing Medical University) were used. The femurs and tibias were harvested and cleaned of surrounding muscle and connective tissue. The bone ends were removed, and the bone marrow was gently flushed out using F‐12 medium (DMEM/F12, obtained from Gibco). The collected bone marrow suspension was then cultured in an incubator at 37 °C with 5% CO_2_, with the medium being changed every 2–3 days to maintain a viable cell culture environment. Before experiments, the cells were characterized by flow cytometry to confirm their surface‐specific markers and purity. To explore how ultrasound affects the acoustic–electric couplers, samples were exposed to different ultrasound parameters, followed by total RNA extraction for RNA – Seq (Shanghai Majorbio Bio–Pharm Technology Co., Ltd., Novaseq X Plus system, USA). The raw data underwent processing with the R programming language to detect DEGs. The criteria for DEGs were *p*–value ≤ 0.05 and fold change > 2. Then, GO analysis was performed to clarify the biological functions of these DEGs, and KEGG pathway analysis was used to identify the enriched signaling pathways.

### Biocompatibility and Osteogenic Verification

BMSCs were cultivated on sterilized short–fiber scaffolds. A series of evaluations performed included live/dead staining, cytoskeleton staining, and CCK–8 proliferation assays, to investigate BMSCs' adhesion, proliferation, and cytotoxicity. The live/dead staining employed the calcein – AM and propidium iodide kit from Beyotime. Cell viability was evaluated by capturing fluorescence images with a Leica confocal microscope. The CCK–8 method was applied to determine cytotoxicity during cell proliferation. To explore the adhesion and morphological changes of BMSCs on the scaffolds, cytoskeleton staining (Beyotime) and SEM were utilized. After 4 days of culture, samples were fixed with 4% paraformaldehyde and permeabilized with 0.1% Triton X–100. Subsequently, cells were stained with 5 µg mL^−1^ Alexa Fluor 594 phalloidin and 10 µg mL^−1^ DAPI to label actin and nuclei, respectively, and observed under a fluorescence microscope (Leica, Japan). In addition to fluorescence staining, SEM was used to assess cell adhesion and morphological changes. After 24 h of culture, cells on the surface were fixed with 2.5% glutaraldehyde for 2 h, followed by gradient dehydration and freeze‐drying, and then observed using SEM (Sirion 200, FEI). The experimental steps related to osteogenesis refer to the previous articles of the research group.^[^ ^24^ ^]^ The in vitro experiments were designed to simulate the “sequential” regeneration process of “antibacterial first, then osteogenesis” in infectious bone defects, using the same sample and simulated physiological environment (α‐MEM medium, 37 °C, 5% CO_2_) to avoid environmental interference. Osteogenic stage: After confirming the effective antibacterial effect, replace the culture medium with α‐MEM medium containing osteogenic inducers. Adjust the ultrasound stimulation parameters and detect osteogenic indicators (alkaline phosphatase (ALP) activity, mineralization nodule formation). Ultrasound irradiation was performed for five days a week, with a two‐day rest period. The ultrasound parameters were then adjusted to 1.0 MHz, 0.5 W cm^−^^2^, and 50% duty cycle.

### siRNA Transfection, Nuclear and Cytoplasmic Protein Extraction, and Immunofluorescence

Synthesis of TRPV4–specific siRNA and negative control siRNA was carried out by Tuoran (Shanghai, China). For transfection, cells at 70–80% confluence in 6–well plates were used. TRPV4 siRNA (100 nm), Lipofectamine 3000, and Opti–MEM were mixed and incubated with the cells for 48 h. The isolation of nuclear and cytoplasmic proteins from experimental samples in accordance with the manufacturer's standard protocol (Thermo Fisher Scientific, USA). The extraction process was meticulously controlled to ensure the high purity and integrity of the separated protein fractions. To verify the separation efficiency, GAPDH was utilized as a cytoplasmic protein marker, and histone 3 (H3) was employed as a nuclear protein marker. Western blotting was conducted to detect the expression levels of these marker proteins in each fraction, thus evaluating the accuracy and reliability of the extraction process. The experimental steps related to immunofluorescence and western blotting refer to the previous articles of the research group.^[^ ^24^ ^]^

### Fura‐2 Single‐Cell Ca^2^⁺ Imaging

BMSCs grown on cell culture slides were washed with Tyrode's solution containing 1.8 mm Ca^2^⁺ and BDM. Subsequently, the cells were incubated with a mixture of 2.5 µm Fura‐2‐AM and 0.05% Pluronic F‐127 at room temperature for 30 min.^[^ ^55^ ^]^ Next, the cell‐coated slides were mounted on an inverted microscope equipped with a CoolSNAP CCD camera and a Lambda XL light source. The fluorescence signals (340/380 ratio) from the cells were recorded and analyzed using MetaFluor fluorescence ratio imaging software and a 20× objective lens.

### Ca^2^⁺ Spark and Wave Measurements

BMSCs were loaded with 2 µm Fluo‐4 AM fluorescent probe and incubated at 37° C for 30 min, followed by an additional 10‐min de‐esterification process. Fluorescence signals were acquired in line‐scan mode using a 488 nm laser on a confocal microscope.^[^ ^56^ ^]^ Line‐scan images of Ca^2^⁺ sparks were analyzed automatically using ImageJ software and Origin 9.0.

### Bacterial Sequencing and Antibacterial Experiments

Bacterial samples were treated with ultrasound at various intensities (0, 0.5, 1, and 1.5 W cm^−^^2^). After treatment, the samples were collected, centrifuged, and frozen in liquid nitrogen before RNA sequencing. The sequencing procedure was the same as the cell part. The analysis standards and procedures followed those described for cellular sequencing. To further evaluate antibacterial efficacy, the test samples were co‐cultured with Staphylococcus aureus and Escherichia coli. After ultrasound treatment (parameters: 1.0 mHz, 1.5 W cm^−^^2^, 50% duty cycle), bacterial suspensions were diluted, and 100 µL of the diluted suspension was evenly spread onto Luria Bertani (LB) agar plates, followed by overnight incubation. Colony counts were determined using ImageJ software, and bacterial viability was assessed using the following formula: C = B∖A×100%. In this formula, C denotes antibacterial efficiency, A represents the number of colonies in the control group, and B stands for the number of colonies in the experimental group. In another set of experiments, different samples were co‐cultured with bacteria on LB agar plates. After ultrasound treatment, the plates were incubated for an additional 24 h. The inhibitory zone effects were observed and statistically analyzed using ImageJ software. Following ultrasound treatment, the culture medium was removed, and bacteria were stained with the LIVE/DEAD BacLight Kit (Beyotime, China) for 15 min. Dark‐field microscopy was used to assess antibacterial effects, and further observations were made using a fluorescence microscope (Leica, Japan). Antibacterial stage: Place Staphylococcus aureus together with electro‐sensitive heterotypic short fibers in an α‐MEM culture medium without osteogenic inducers for cultivation. Apply ultrasound stimulation (power density 1.5 watts/square centimeter, frequency 1mHz) for 20 min to activate the acoustic‐electrical coupling effect of the fibers. The antibacterial rate was measured by the colony counting method.

### In Vivo Model Establishment, Antibacterial Experiments, and Imaging Assessment

The animal experimental procedures were approved by the Animal Research Committee of Chongqing Medical University and were strictly followed by the NIH guidelines. The ethical number was IACUC‐CQMU‐2023‐0434. The experimental animals comprised Sprague–Dawley rats weighing 250–300 g and 6‐week‐old male BALB/c nude mice. All animals were kept in a specific pathogen‐free (SPF) environment. The animals were randomly divided into four groups: Control, US@BaTiO_3_@Fe group, Fibers group, and US@Fibers group. During the experiment, rats were anesthetized via intraperitoneal injection of a 2.0% sodium pentobarbital/saline solution (30 mg kg^−1^). After anesthesia induction, the hair on the femur was removed, and the femur was exposed. A bone defect with a 3 mm diameter was created in the femur using a dental drill. Subsequently, a bacterial suspension containing Staphylococcus aureus (concentration of 10⁹ CFU mL^−1^, 10 µL) was implanted into the femoral defect site, followed by the insertion of BaTiO_3_@Fe or graphene‐modified fiber scaffolds, or unmodified fiber scaffolds. Ultrasound treatment was initially administered at 1.0 mHz, 1.5 W cm^−^^2^, and 50% duty cycle for 20 min, five days per week with a two‐day rest period, for a total of four weeks. The ultrasound parameters were then adjusted to 1.0 mHz, 0.5 W cm^−^^2^, and 50% duty cycle. (Figure S12, Supporting Information). To track the antibacterial effects, Staphylococcus aureus labeled with indocyanine green (ICG) fluorescence was used. In vivo imaging was performed using an in vivo imaging system (IVIS) before and after ultrasound treatment in different target nude mice, and the results were analyzed. Bone tissue samples from the surgical sites were collected at 2 and 4 weeks post‐surgery (six rats per group) and processed into paraffin‐embedded sections. Following fixation in 70% ethanol for a period of three days, micro–CT scanning (SkyScan1172 Ex – Vivo MicroCT, Belgium) was performed on the rat skulls to achieve 3D reconstruction. The analyzed parameters included BV/TV, bone density, Tb.Th, Tb.N, and Tb.Sp.

### Histological Section Analysis

To assess osteogenic mineralization capacity and antimicrobial performance in living organisms, surgical specimens were harvested at postoperative weeks 2 and 4 for histological processing. Following extraction, bone segments underwent immersion in 4% paraformaldehyde solution (pH 7.4) maintained at 4 °C throughout the 7‐day fixation protocol, with solution renewal performed minimally twice throughout fixation. Subsequent to decalcification procedures utilizing established protocols from prior investigations, specimens were embedded in paraffin and sectioned into 5‐µm slices using a rotary microtome. These tissue sections served as substrates for histomorphometric evaluation through H&E staining, collagen matrix visualization via Masson's trichrome technique, and molecular characterization through immunofluorescence assays. The immunolabeling protocol consisted of sequential phases: Femoral sections underwent room temperature desiccation followed by membrane permeabilization using 0.1% Triton X‐100/PBS solution (15 min incubation). Non‐specific binding sites were blocked with 5% caprine serum (30 min, ambient conditions). Primary immunoreagents targeting Collagen I and osteopontin (OPN), prepared in 5% caprine serum carrier solution, were subjected to overnight incubation at 4° C in a humidified chamber. Following three PBS washing cycles (5 min each), fluorochrome‐conjugated secondary antibodies were applied under light‐protected conditions (RT, 1 h). Nuclear architecture was delineated through DAPI counterstaining (10 µg mL^−1^, 2 min exposure). Mounted specimens preserved with polyvinyl alcohol‐based sealant underwent confocal laser scanning microscopy analysis. Fluorescence quantification was conducted through standardized digital image analysis protocols using ImageJ software.

### Statistical Analysis

The experimental data were expressed as the mean ± standard deviation, based on at least three independent experimental replicates. In terms of statistical analysis, Origin Software and GraphPad Prism 8.0 were employed. For comparisons between two groups, Student's *t*‐test was used, whereas one‐way or two‐way ANOVA was applied for comparisons among multiple groups. A *p*‐value of less than 0.05 was regarded as statistically significant. (^*^ *p* < 0.05, ^**^ *p* < 0.01, ^***^ *p* < 0.001, ^****^ *p* < 0.0001; data were presented as mean ± SD, n = 3 or 6. NS, no significant difference).

## Conflict of Interest

The authors declare no conflict of interest.

## Author Contributions

X.H., F.W., P.X. and Z.Y. contributed equally to this work. X.H., F.W., Z.Y., and P.X. led the conception, design, and writing of the whole study, and played a decisive role in controlling the research direction and solving key problems. They were the core contributors of this study. W.C., J.W., and D.B. had given key guidance on the overall idea of the study, and had made outstanding contributions to the deepening of the viewpoint and the improvement of the quality of the article. They were the key leaders of the study. Z.H. and Z.W. were responsible for most of the experimental execution and data collection. Their rigorous experimental operation and data analysis provided reliable data support for the study, and they were important performers of the study. A.J. and J.T. were mainly responsible for the preparation of part of the experimental samples and the preliminary processing of the preliminary data, and made significant contributions to the preliminary preparation of the study.

## Supporting information



Supporting Information

Supplemental Movie 1

Supplemental Movie 2

Supplemental Movie 3

Supplemental Movie 4

## Data Availability

The data that support the findings of this study are available from the corresponding author upon reasonable request.
