# Supplementary material for: Electrosensitive Heterogeneous Short Fibers via Acousto‐Electric Coupling for Sequential Bone Regeneration in Infectious Defects
Source: Adv Sci (Weinh). 2025 Oct 13;12(48):e14174. doi: 10.1002/advs.202514174 (PMC12752602; doi:10.1002/advs.202514174)
Supplement: Supplementary file 1 — Supporting Information [file ADVS-12-e14174-s001.docx]

Supplementary Materials

**Electrosensitive Heterogeneous Short Fibers via Acousto-Electric Coupling for Sequential Bone Regeneration in Infectious Defects**

Xiaoyu Han, Fan Wang , Pengcheng Xiao , Zheng Yang , Mingyue Liu, Zeyu Han, Zijie Wang , Anan Jiang, Jindong Tan, Juan Wang *, Wenguo Cui *, Dingqun Bai *

X. Han, P. Xiao, Z. Yang, Z. Wang, A. Jiang, J. Tan, D. Bai.

Department of Rehabilitation Medicine, Key Laboratory of Physical Medicine and Precision Rehabilitation of Chongqing Municipal Health Commission, The First Affiliated Hospital of Chongqing Medical University, No.1 Youyi Road, Yuzhong District, Chongqing, 400016, China.

F.Wang,M.Liu, Z.Han, J.Wang, W.Cui.

Department of Orthopaedics, Shanghai Key Laboratory for Prevention and Treatment of Bone and Joint Diseases, Shanghai Institute of Traumatology and Orthopaedics, Ruijin Hospital, Shanghai Jiao Tong University School of Medicine, 197 Ruijin 2nd Road, Shanghai 200025, People's Republic of China.

X. Han, P. Xiao, Z. Yang, Z. Wang, A. Jiang, J. Tan, D. Bai.

State Key Laboratory of Ultrasound in Medicine and Engineering, Chongqing Medical University, Chongqing, 400016, China.

* Corresponding author. Email: [baidingqun@hospital.cqmu.edu.cn](mailto:baidingqun@hospital.cqmu.edu.cn) (D.Q. Bai), [juanwang1006@126.com](mailto:juanwang1006@126.comg) (J.Wang); wgcui80@hotmail.com (W.G. Cui)

**Figure S1.**


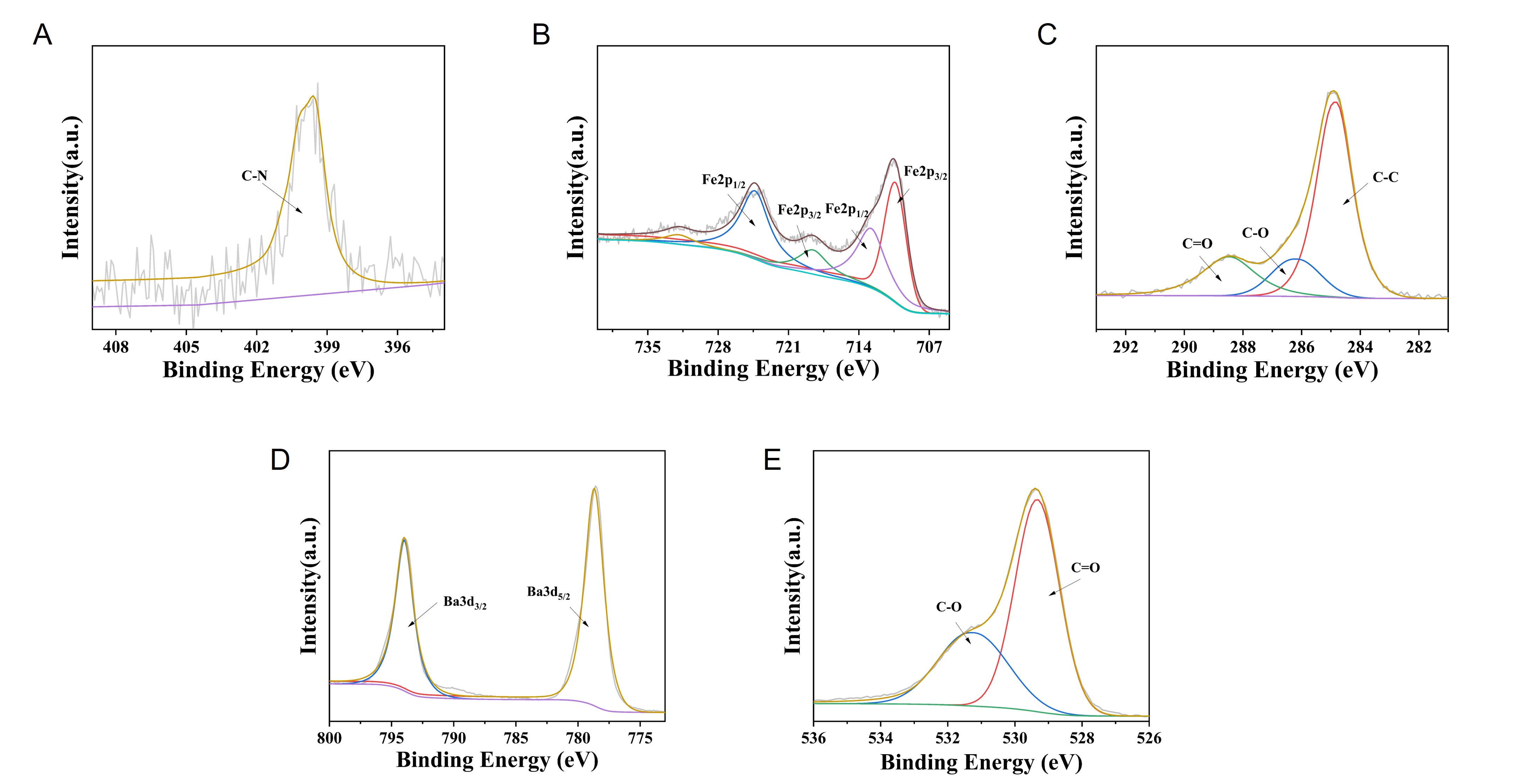


**Figure S1.** The XPS results of electrosensitive heterogeneous short fibers.

**Figure S2.**


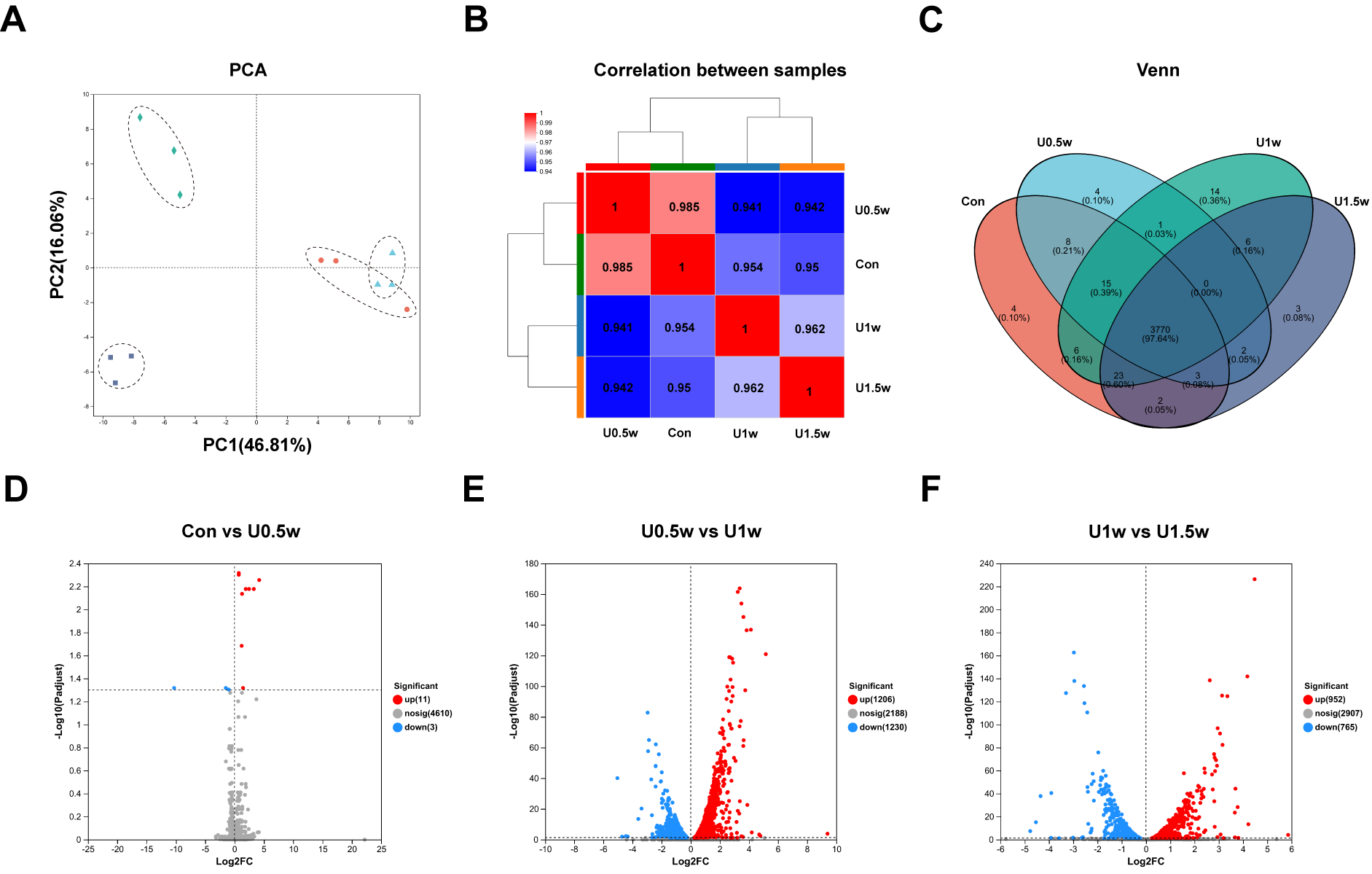


**Figure S2.** The quality control data of the sequencing results.

**Figure S3.**
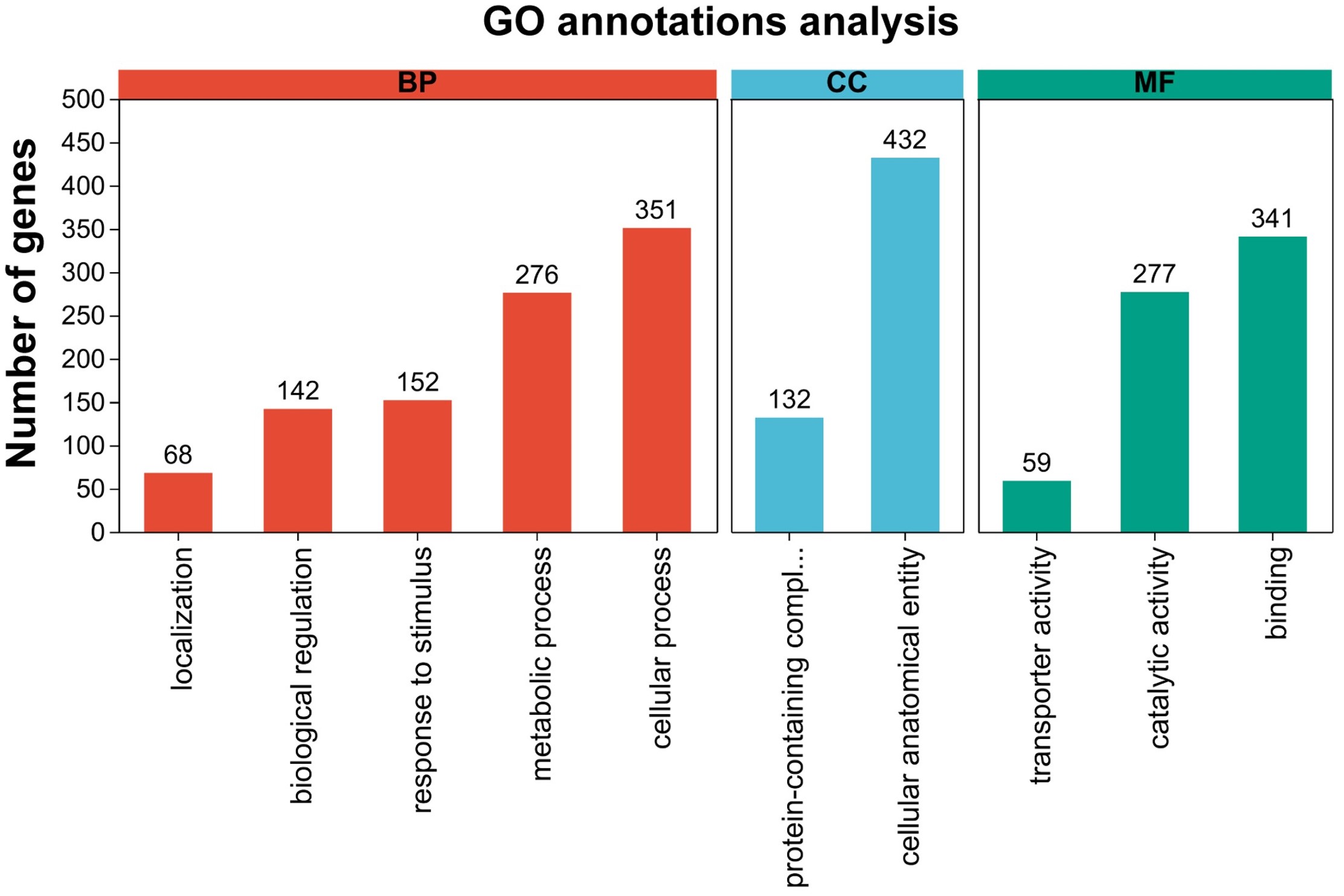


**Figure S3.** Gene Ontology (GO) enrichment analysis.

**Figure S4.**


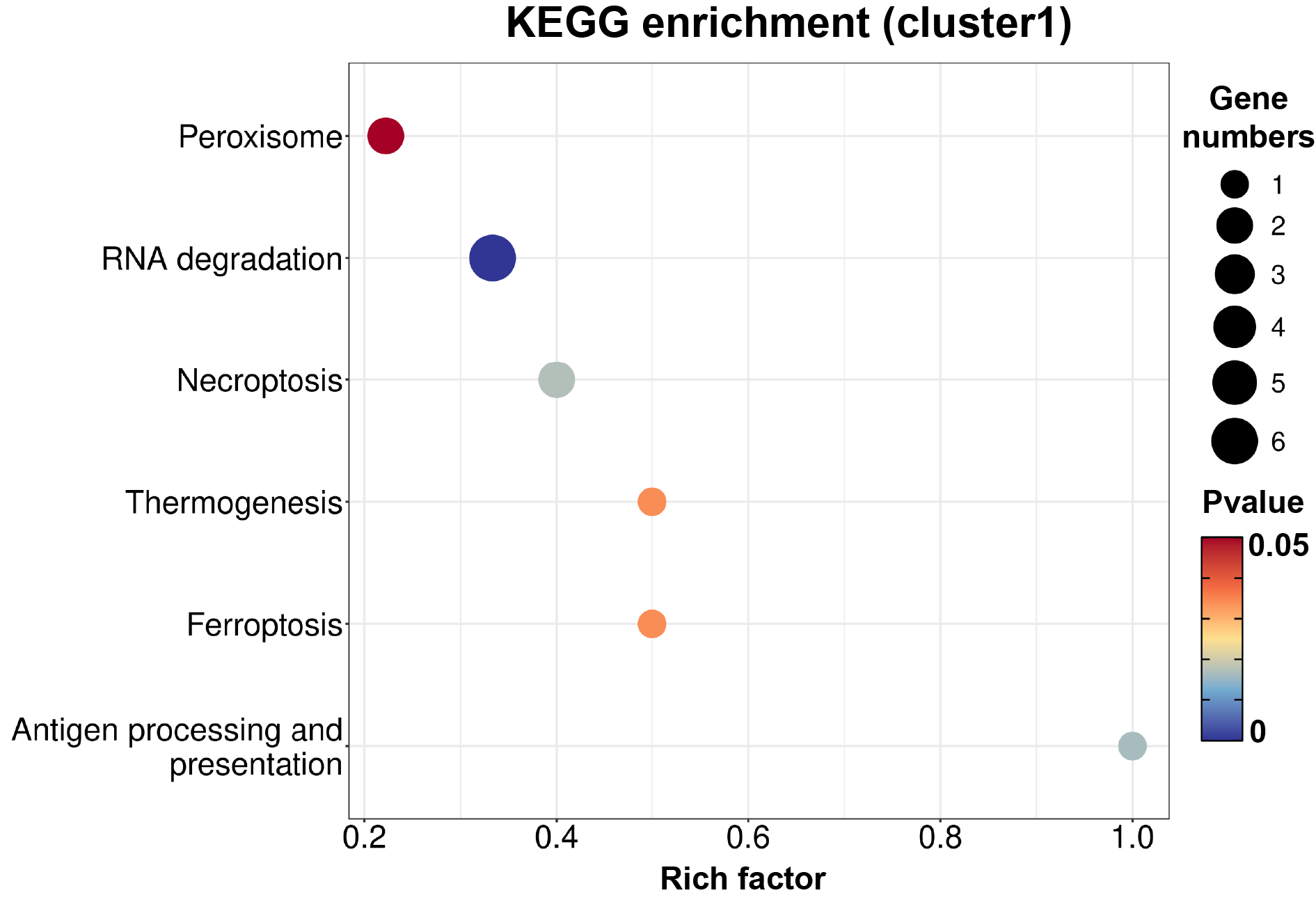


**Figure S4.** KEGG enrichment analysis (1 W vs. 1.5 W).

**Figure S5.**
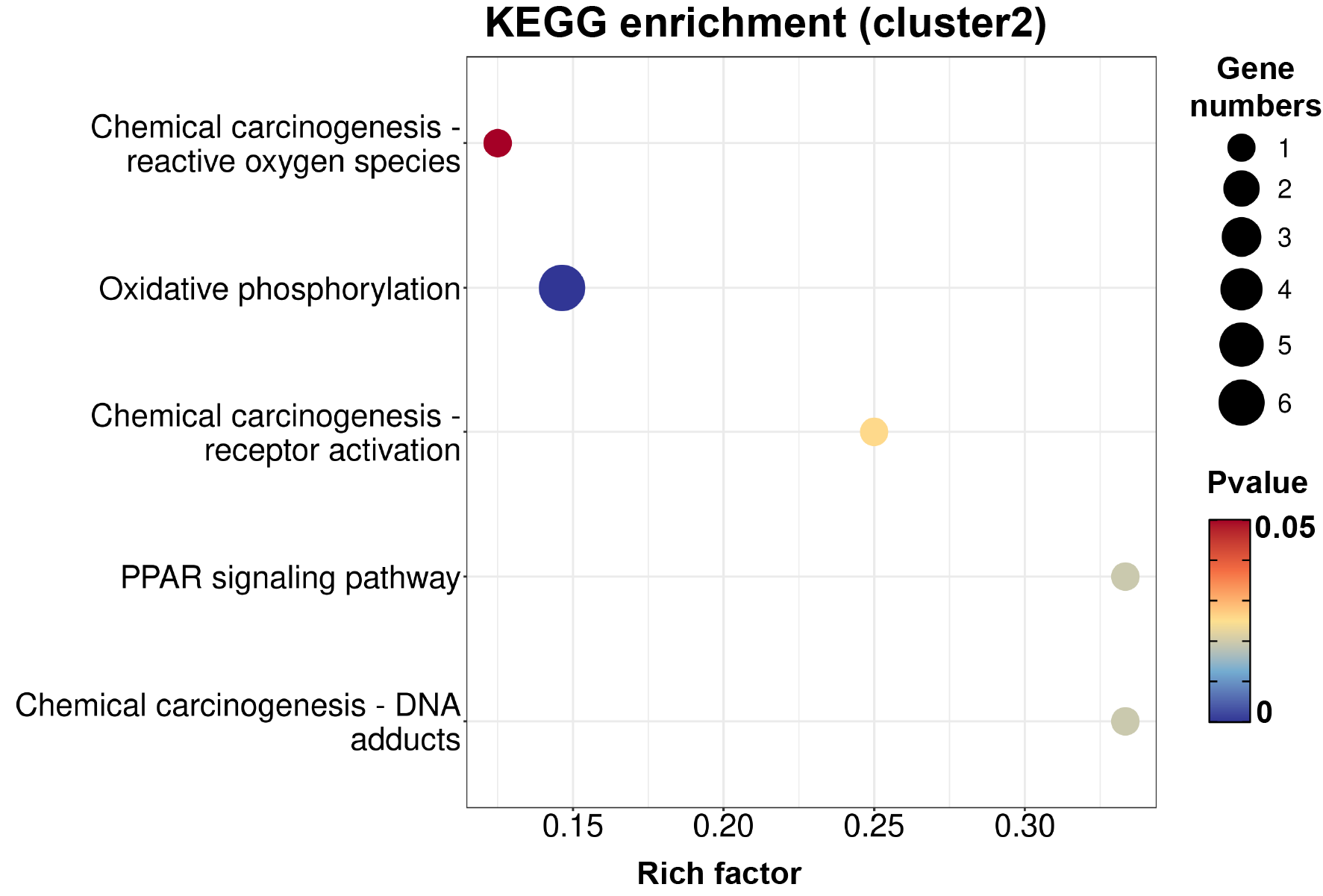


**Figure S5.** KEGG enrichment analysis (0.5 W vs. 1 W).

**Figure S6.**
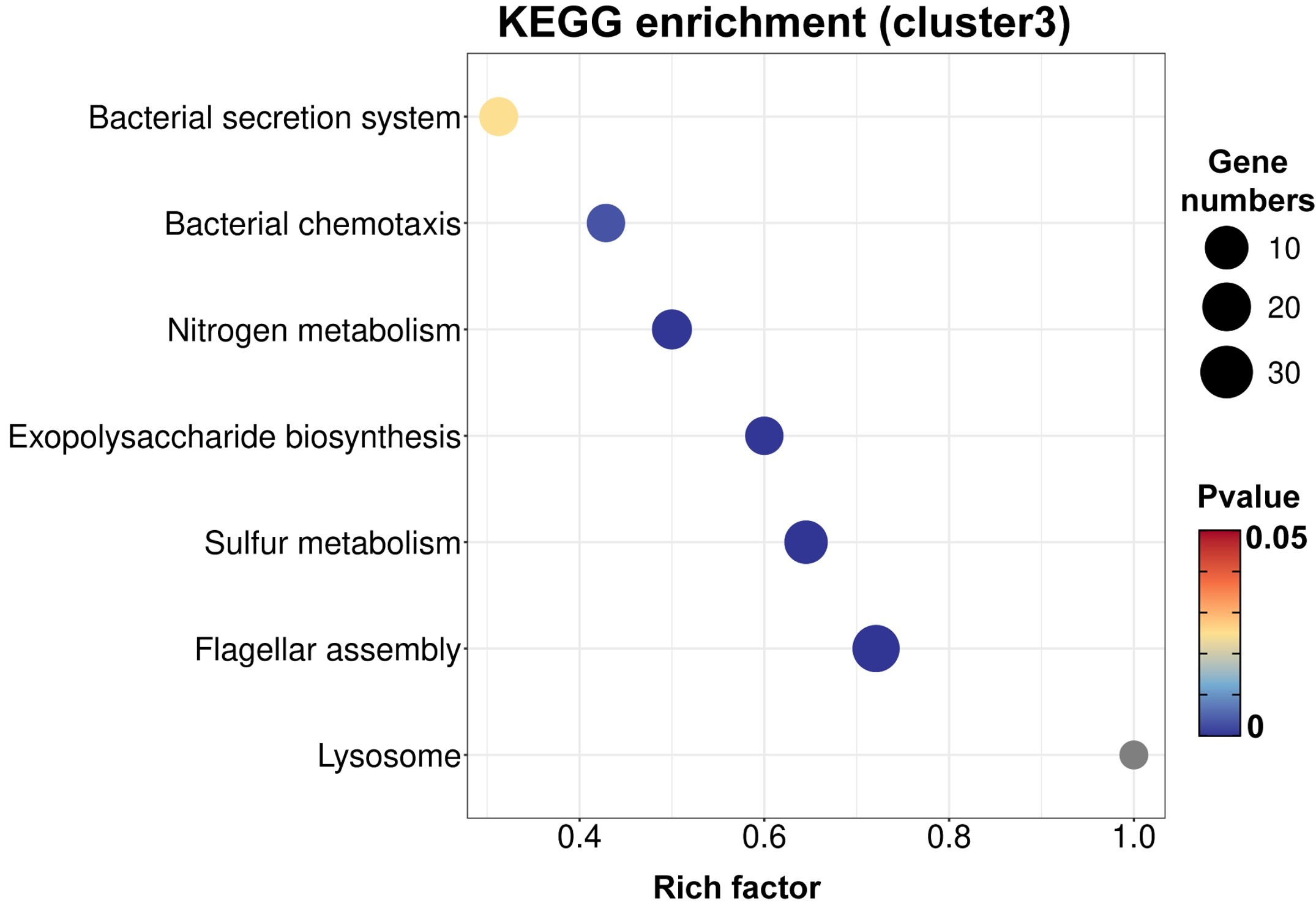


**Figure S6.** KEGG enrichment analysis (Control vs. 0.5 W).

**Figure S7.**


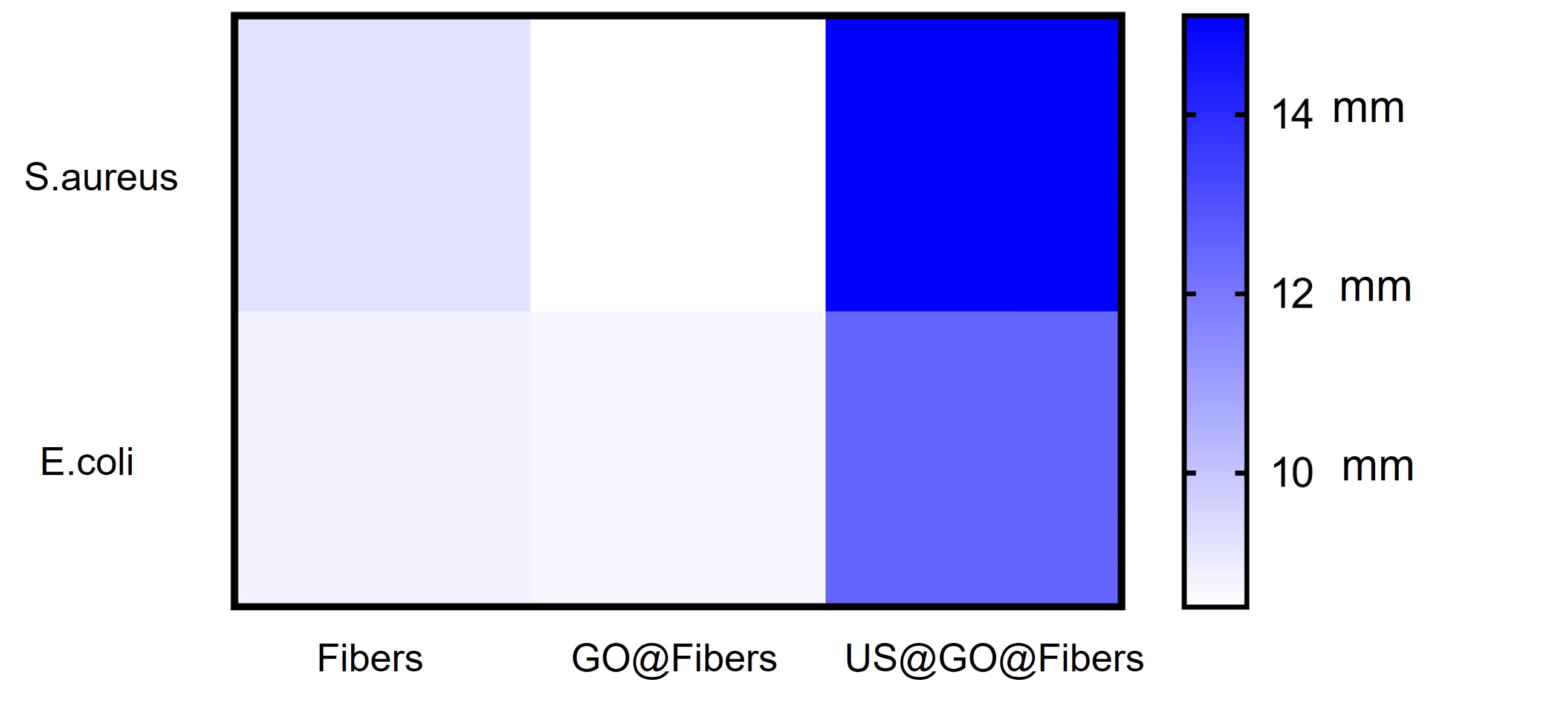


**Figure S7.** Statistical diagram of experimental data of bacteriostatic zone.

**Figure S8.**


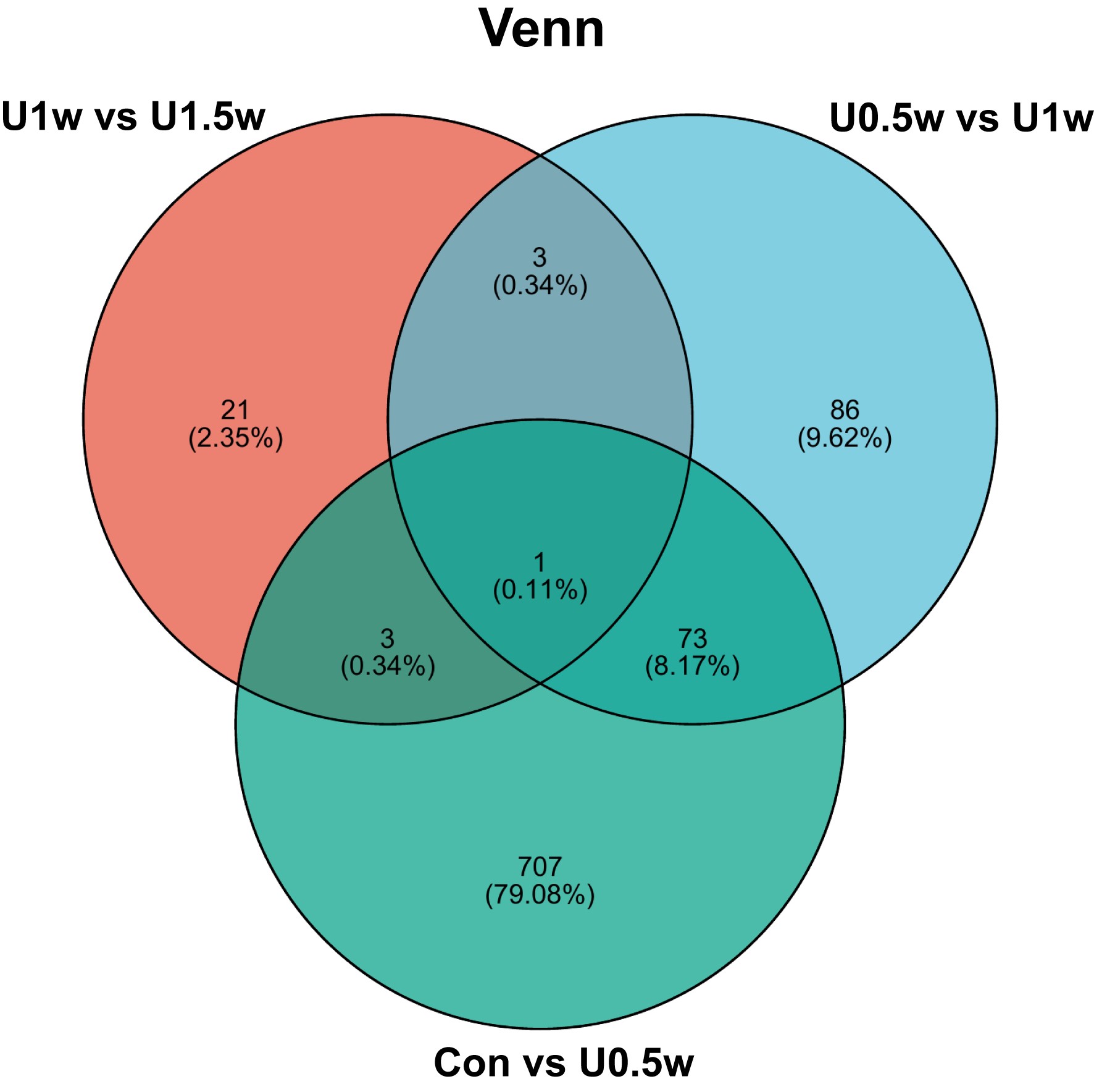


**Figure S8.** Correlation heat maps of gene sequencing under different ultrasonic parameters.

**Figure S9.**


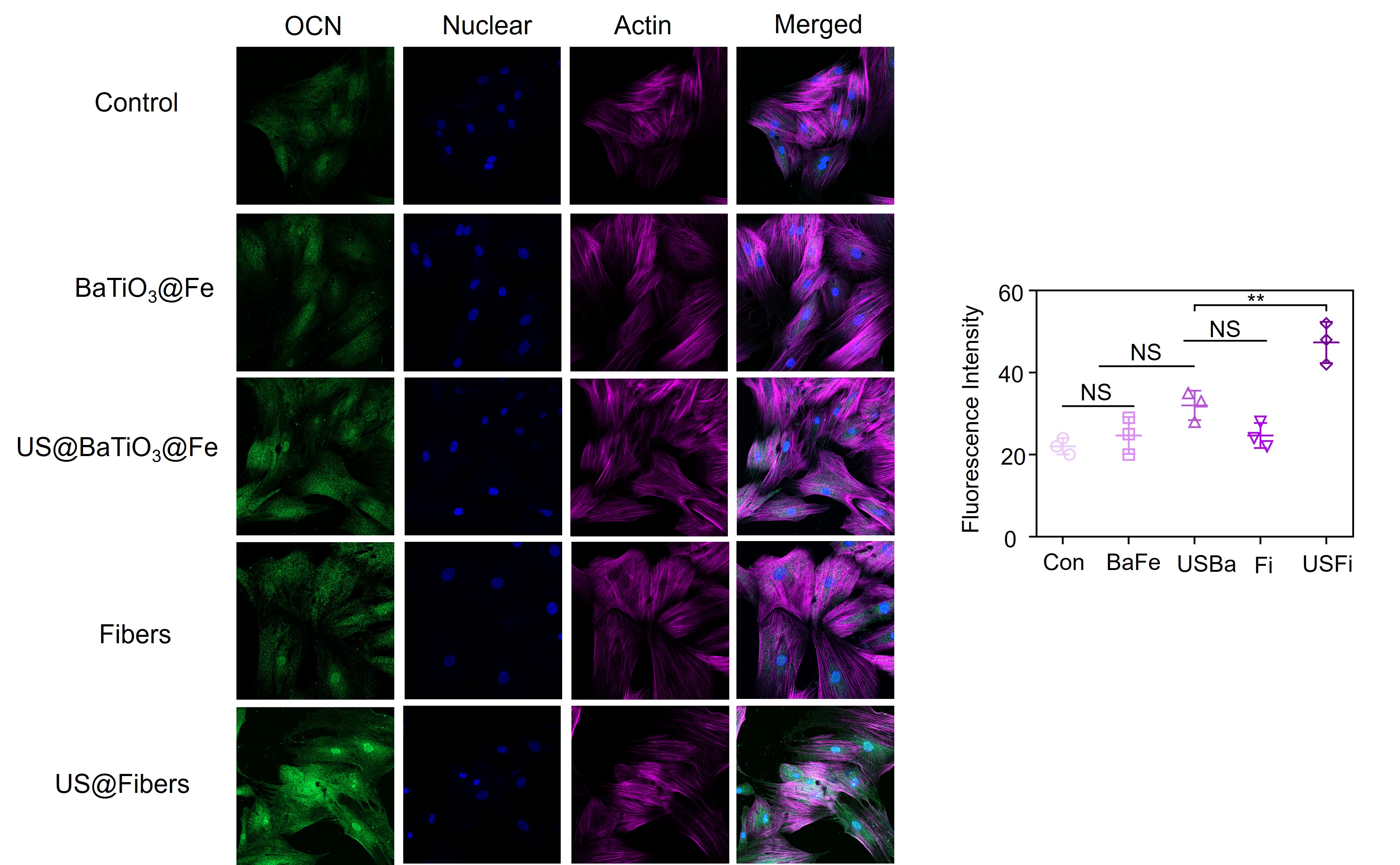


**Figure S9.** Cellular immunofluorescence of OCN.

**Figure S10.**


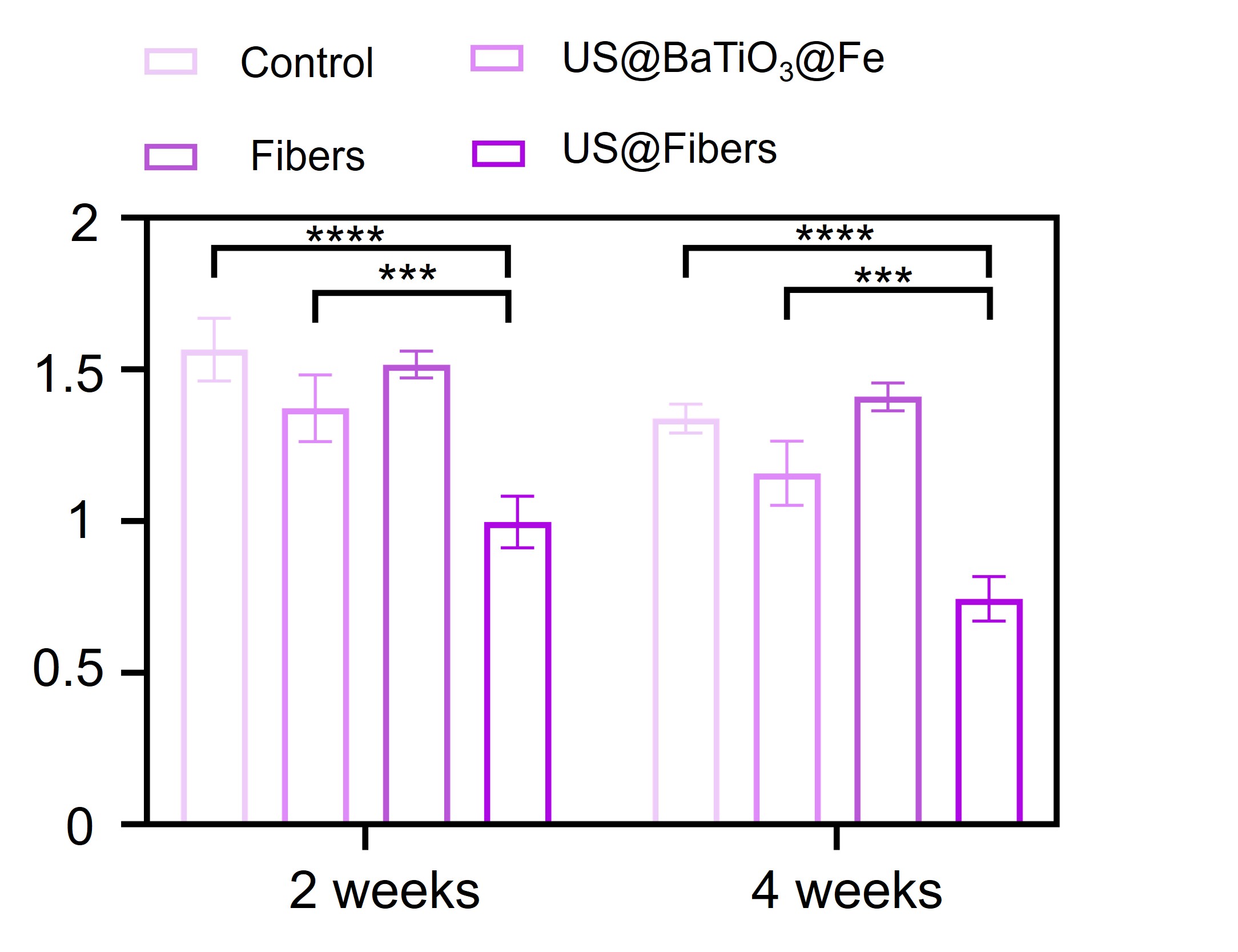


**Figure S10.** The result statistics of Tb.Sp.

**Figure S11.**


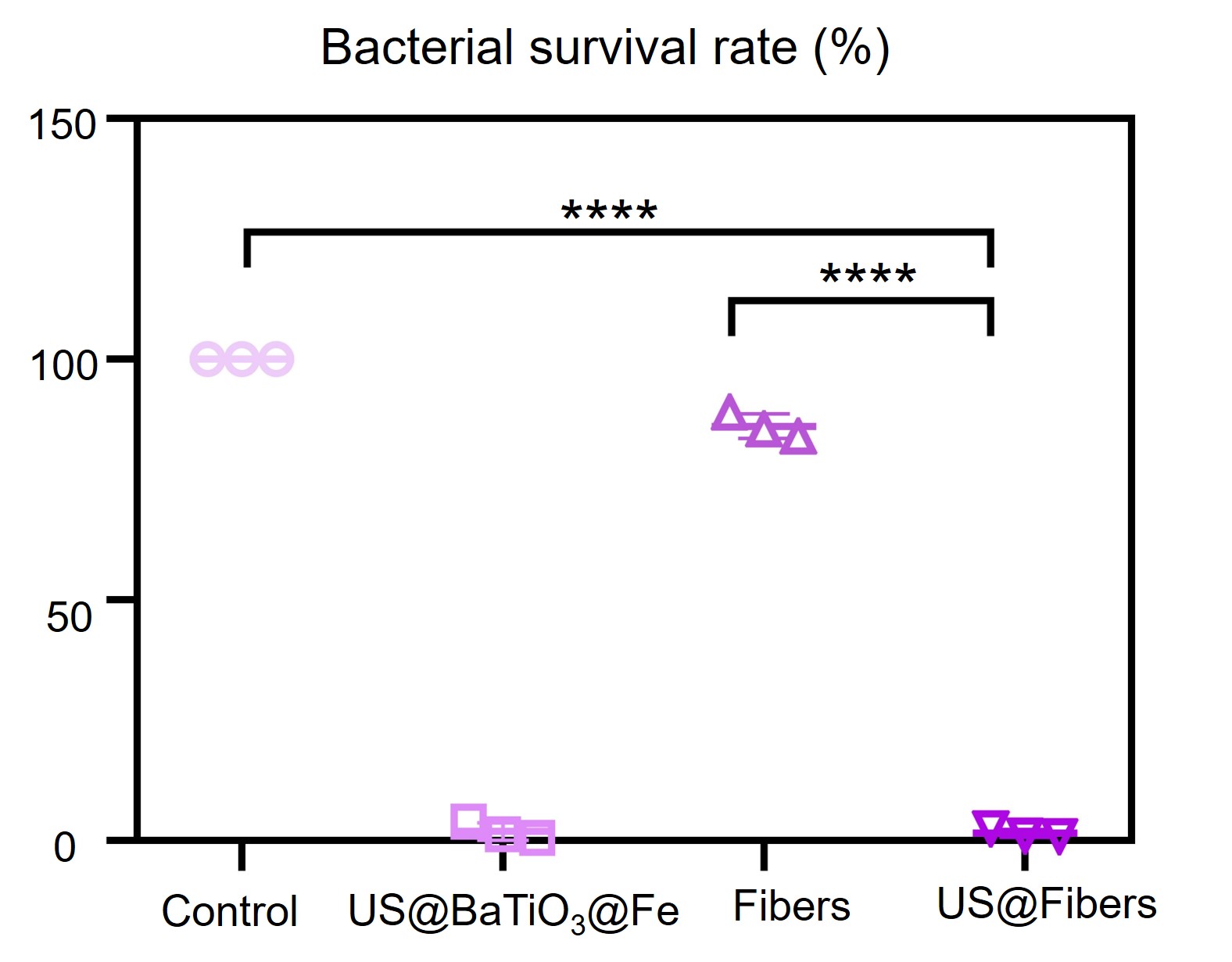


**Figure S11.** Statistical map of antibacterial *in vivo*.

**Figure S12.**


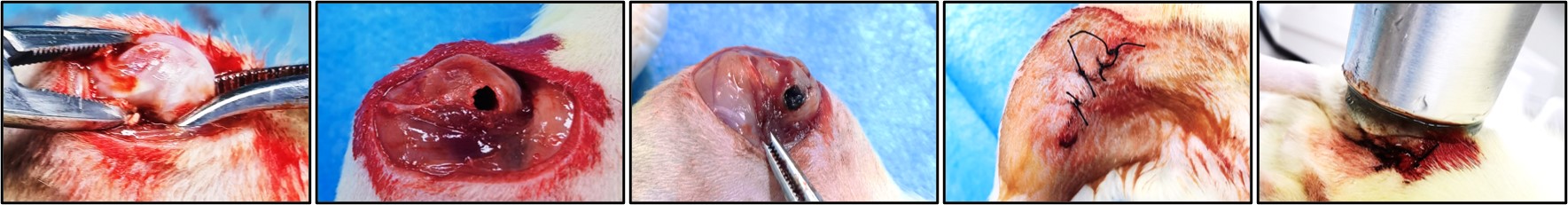


**Figure S12.** Modeling process of femur defect in SD rats.

**Movie 1-4.** Real-time imaging of calcium ions under different parameters.
